# Second harmonic generation microscopy reveals the spatial orientation of glutamine-potentiated liver regeneration after hepatectomy

**DOI:** 10.1097/HC9.0000000000000640

**Published:** 2025-03-07

**Authors:** Chih-Chieh Yen, Chia-Sheng Yen, Hung-Wen Tsai, Matthew M. Yeh, Tse-Ming Hong, Wen-Lung Wang, I-Ting Liu, Yan-Shen Shan, Chia-Jui Yen

**Affiliations:** 1 Institute of Clinical Medicine, College of Medicine, National Cheng Kung University, Tainan, Taiwan; 2 Department of Oncology, National Cheng Kung University Hospital, College of Medicine, National Cheng Kung University, Tainan, Taiwan; 3 Division of General Surgery, Department of Surgery, Kaohsiung Veterans General Hospital, Kaohsiung, Taiwan; 4 Department of Nursing, Meiho University, Pingtung, Taiwan; 5 Department of Pathology, National Cheng Kung University Hospital, College of Medicine, National Cheng Kung University, Tainan, Taiwan; 6 Department of Laboratory Medicine and Pathology, University of Washington School of Medicine, Seattle, Washington, USA; 7 Department of Medicine, University of Washington School of Medicine, Seattle, Washington, USA; 8 Department of Surgery, National Cheng Kung University Hospital, College of Medicine, National Cheng Kung University, Tainan, Taiwan

**Keywords:** glutamine, hepatectomy, liver regeneration, second-harmonic generation microscopy, two-photon excitation fluorescence

## Abstract

**Background:**

Glutamine (Gln) is a critical amino acid for energy expenditure. It participates in extracellular matrix (ECM) formation and circulates in the hepatic parenchyma in a spatial-oriented manner. Posthepatectomy liver mass recovery poses a regenerative challenge. However, little is known about the role of Gln in liver regeneration, notably the spatial orientation in the remodeling process. This study aimed to elucidate Gln-potentiated liver regeneration and ECM remodeling after mass loss.

**Methods:**

We studied the regenerative process in hepatectomized mice supplemented with Gln. Second harmonic generation/two-photon excitation fluorescence microscopy, an artificial intelligence–assisted structure-based imaging, was used to demonstrate the spatial-oriented process in a hepatic acinus.

**Results:**

Gln promotes liver mass regrowth through the cell cycle, Gln metabolism, and adipogenesis pathways after hepatectomy. Ornithine transaminase, one of the upregulated enzymes, showed temporal, spatial, and functional correspondence with the regeneration process. Second harmonic generation/two-photon excitation fluorescence microscopy highlighted transient hepatic steatosis and ECM collagen synthesis, predominantly in the portal tract instead of the central vein area. Structural remodeling was also observed in the portal tract area.

**Conclusions:**

Gln promotes liver regeneration through cellular proliferation and metabolic reprogramming after hepatectomy. Using structure-based imaging, we found that Gln potentiated hepatic steatosis and ECM collagen deposition predominantly in the portal tract area. These results highlighted the spatial orientation and mechanistic implications of Gln in liver regeneration.

## Graphical abstract

**Figure:**
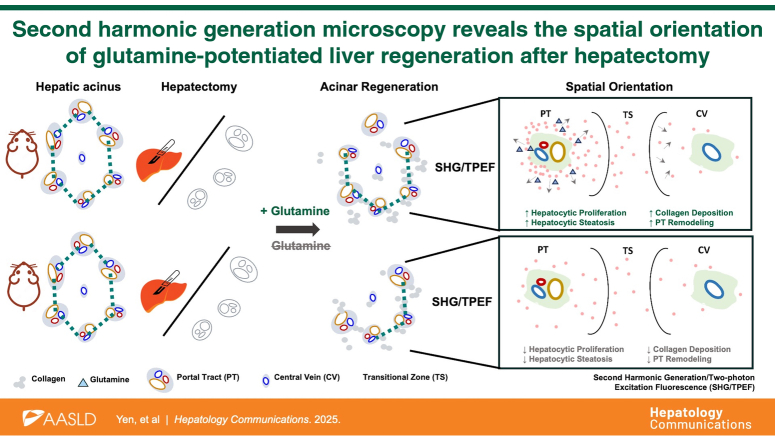

## INTRODUCTION

Extended hepatectomy plays a crucial role in various therapeutic purposes. However, with a remaining liver parenchyma of 20%–30% of the initial volume, it is a high-risk procedure with risks to induce hepatic failure.^1^,^2^,^3^ Hepatic regrowth poses a regenerative challenge to the remnant liver in terms of proliferative and metabolic demands.^4^ Notably, liver regeneration is a well-orchestrated process involving hepatocytic proliferation, repopulation, and structural remodeling.^5^,^6^,^7^ Functional hepatic zonation confers spatial transformation and physiological coordination in a hepatic acinus.^8^ Interestingly, the resemblance between rodents and humans in liver regeneration provides a strong rationale for investigating the process in mice, since such harmful and risky experiments are difficult to replicate in clinical studies.^9^ Therefore, elucidating the orchestration of the liver regeneration process demonstrates good clinical value and research potential.

Glutamine (Gln) is a critical and abundant nonessential amino acid that participates in cellular metabolism, energy expenditure, and extracellular matrix (ECM) formation.^10^,^11^,^12^ Predominantly in the liver, Gln plays an important role in nitrogen balance, amino moiety donation, and ammonia scavenging in the urea cycle.^13^,^14^ Conversely, its metabolism involves a coordinated process following the functional hepatic zonation, also known as “intercellular Gln cycle.”^12^,^15^,^16^ Several studies on rodent hepatectomy revealed that Gln increased the net liver mass regrowth.^17^,^18^ However, the critical spatial orientation has not been fully explored. Since Gln inherently circulates in the hepatic parenchyma according to its functional organization, it is a reasonable choice to be selected as a potentiator to induce the regeneration process in a spatial-oriented manner. In addition, studies concerning liver regeneration encompass 2 major types of injuries: toxin-induced and hepatectomy-induced injuries. While the former could sufficiently induce regenerative stress mimicking extensive and diffuse parenchymal insults, the latter, on the other hand, inherently correlates with distinctive clinical scenarios and purposes, such as healthy living individuals planning for an extended liver mass donation.^8^,^19^

The spatial orientation within the hepatic acinus plays a pivotal role in regeneration. However, this delicate process poses research barriers owing to the limitations of conventional morphological assays. With the help of laser-excited autofluorescence imaging, second harmonic generation/two-photon excitation fluorescence microscopy (SHG/TPEF) provides access to the elucidation of the spatial clues in various pathological conditions in the liver. Xu et al^20^ proposed the quantification of hepatic fibrosis with SHG/TPEF and applied it in animal and human models. Chang et al^21^ further validated it in the automated staging of hepatic fibrosis in patients with metabolic dysfunction–associated steatotic liver disease. While growing evidence has suggested the interplay of hepatic steatosis and fibrosis, recent studies have addressed the methodology, validation, and application of SHG/TPEF in measuring miscellaneous changes in the liver parenchyma, such as fibrosis, inflammation, ballooning, and steatosis in patients with chronic liver diseases through artificial intelligence (AI)-assisted algorithmic approach.^22^,^23^,^24^ To date, such a technique has never been described or applied in the liver regeneration process. Since Gln could be used as a functional potentiator for liver regeneration, it raises a research motive to observe the spatial orientation using a novel imaging method and corresponds to knowledge concerning metabolic shifts and organ remodeling.

The aim of the present study was to elucidate Gln-potentiated liver regeneration and ECM remodeling after mass loss. To address this issue, we studied the regenerative process in hepatectomized mice supplemented with Gln. SHG/TPEF was used to demonstrate the spatially oriented process in the hepatic acinus. These results highlight the utility of novel imaging techniques in assessing subtle changes during liver regeneration and provide spatial clues into the remodeling process, which could transform into a clinical value in patients undergoing extended hepatectomy.

## METHODS

### Animal and cell line

Eight-week-old male specific pathogen-free C57BL/6 mice were purchased from NLABRC. All mice were treated, manipulated, and maintained at the LAC, NCKU. Murine liver specimens were collected according to experimental protocols at specific time points after CO_2_ euthanasia. Repeated tail vein peripheral blood sampling was conducted using a 27-gauze needle at specific time points, with each of 0.1 mL. Human hepatic stellate ce﻿line TWNT-1 was purchased from Bioresource Collection and Research Center and maintained in a 1:1 mixture of DMEM and Ham’s F12 medium (DMEM-F12, Hyclone; GE Healthcare Bio-Sciences Corp.) containing 10% fetal bovine serum (GIBCO; Thermo Fisher Scientific), 5 μg/mL insulin, 5 μg/mL transferrin, and 5 ng/mL selenium in a humidified incubator with 5% CO_2_ at 37°C.

### Seventy percent partial hepatectomy and Gln supplementation

The murine partial hepatectomy (PHx) model was first described by Anderson and Higgins.^25^ We followed the revised protocol described by Mitchell and Willenbring.^26^ The details of the procedure are provided in Supplemental Data S1A, http://links.lww.com/HC9/B889. Briefly, 8-week-old C57BL/6 mice underwent either 70% PHx or sham surgery in the absence of hepatic resection through midline laparotomy (Supplemental Data S2, http://links.lww.com/HC9/B889). The remnant liver weight/body weight ratio of mice receiving PHx is shown in Supplemental Data S3, http://links.lww.com/HC9/B889. l-glutamic acid 5-amide (Gln) was obtained from MilliporeSigma, and mice were fed 0.6% Gln versus PBS in the drinking water starting at 7 days before PHx and another 6 days after Phx. Mice were randomized into 4 groups: (1) sham with Gln supplementation (sham + Gln), (2) sham without Gln supplementation (sham − Gln), (3) PHx with Gln supplementation (PHx + Gln), and (4) PHx without Gln supplementation (PHx − Gln). Remnant livers were collected for further analyses and weighed daily from days 1 to 6 after PHx.

### Immunohistochemistry and immunoblotting

Remnant liver samples were fixed in 4% formalin and embedded in paraffin. Paraffin-embedded blocks were cut into 5 μm sections. Sample slides were subjected to immunohistochemical (IHC) staining, and antigens were retrieved by heating in a buffer solution for 30 minutes, according to the manufacturer’s instructions (Invitrogen). For immunoblotting, total tissue lysates from liver samples were prepared, separated by SDS-PAGE, and transferred to the PVDF membrane (MilliporeSigma). Detailed information on IHC and immunoblotting is provided in Supplemental Data S1B, C, http://links.lww.com/HC9/B889, respectively.

### TissueFAXS imaging quantification

Sample slides were prepared from formalin-fixed paraffin-embedded sections and stained using IHC. Images were captured from the TissueFAXS platform (TissueGnostics) and analyzed by TissueQuest software (TissueGnostics) in the Optical Image Core Laboratory, Clinical Medicine Research Center, National Cheng Kung University Hospital. The analytical information is provided in Supplemental Data 1D, http://links.lww.com/HC9/B889.

### Gene expression microarray and gene set enrichment analysis

Liver samples were prepared for mRNA expression microarray from day 1 to day 6 after PHx by Welgene Biotech Co. Ltd. Briefly, microarray analyses were performed using the SurePrint G3 GeneChip Mouse Gene Express V2 Array Kit (Agilent) according to the manufacturer’s protocols. Detailed information on the mRNA expression microarray is provided in Supplemental Data S1E, http://links.lww.com/HC9/B889. The analytic workflow of the processed data, quality assessment, and gene set enrichment analysis are shown in Supplemental Data S5, http://links.lww.com/HC9/B889.

### Quantitative real-time reverse transcriptase-PCR

A total of 1 μg of RNA was used to synthesize cDNA using an M-MLV reverse transcriptase kit (Thermo Fisher Scientific). The mRNA levels of mouse *gapdh*, *actb*, *cdkn2a*, *cdk1*, *cps1*, *oat*, and *glul* were detected using the validated specific primers/probes of TaqMan Gene Expression Assays (Thermo Fisher Scientific) and TaqMan Universal PCR Master Mix (Thermo Fisher Scientific) according to the manufacturer’s instructions. Real-time PCR assays were performed using a StepOne Real-Time PCR System (Applied Biosystems). The signals for inducible cellular mRNAs were normalized to the mRNA signal of the housekeeping gene, *actb*. The target mRNA levels were compared and normalized according to the experimental groups or conditions.

### Second harmonic generation/two-photon excitation fluorescence microscopy imaging

Liver specimens were processed for image acquisition using SHG/TPEF (Genesis system; Histoindex Pte.).^27^,^28^ Briefly, SHG (390 nm) captured the architectural features of ECM collagen fibrils in the unstained slides through laser excitation (780 nm), and TPEF signals (550 nm) visualized the hepatocytes, inflammatory cells, and fat vacuoles.^20^ Each image was acquired at ×20 magnification with a resolution of 512 × 512 pixels for visualization, in which a 200 × 200 μm^2^ RGB image tile was constructed. To maximally encompass the whole-slide image, 5 × 5 image tiles (1 mm × 1 mm) were taken as a unit of the ROI. ROIs (10–15) were calculated on each slide as an average to demonstrate collagen deposition in the extracellular space and steatotic hepatocytes, which generated a total of 10–15 mm^2^ final acquisition size in each sample (10–15 × 1 mm × 1 mm).

The regions of liver tissue and collagen were identified from TPEF and SHG channels using Otsu’s automatic threshold method. The SHG signals outside of the liver specimen and residuals were identified as noise and removed from the analysis. Furthermore, an AI-based algorithm divided the hepatic parenchymal structures into 5 independent areas from TPEF signals, including the portal tract (PT), periportal (PP), transitional, pericentral, and central vein (CV) areas, through a digital image processing method and Classification And Regression Tree model for object identification and segmentation.^27^ The PP and pericentral areas were set at 100 μm from the PT and CV, respectively, and the region in between was the transitional area. Collagen deposition within the hepatic acinus was quantified separately based on the percentage of total collagen in each area, with the signals acquired through SHG. Hepatic steatosis was measured by the size and density of triacylglycerol vacuoles accumulating in the hepatocytes. Since all liver sections were deparaffinized, the fluorescence emitted by the lipid vacuoles from the hepatocytes was a “hollow” where no emittance was generated. The absorptiometry thereby revealed the lipid vacuoles as dark signals in contrast to the red ones from the cytoplasm of hepatocytes. The scalable parameters were, therefore, calculated by the AI-based image algorithm. Lipid vacuoles were distinguished from other tissue cavities, such as vessels, bile ducts, sinus space, and tissue cracks, based on the morphological features of the cavities and the surrounding collagen. Steatotic hepatocytes were quantified as a proportion of the total hepatocytes. Fluorescent or transformed images were obtained for visual purposes. The spatial quantification results were compared between the PHx + Gln and PHx − Gln mice. The detailed algorithmic process for the SHG/TPEF is provided in Supplemental Data S1F, http://links.lww.com/HC9/B889.

### Statistical analysis

All numerical data are presented as mean ± SEM. The results of each experiment were obtained from at least 3 independent replicates. Student *t* test and 1-way ANOVA with post-hoc testing by the Tukey honestly significant difference test were used to compare continuous variables. Specific considerations for pairwise multiple comparisons and time-dependent repeated measures under a mixed-effect model are described in Supplemental Data S1G, http://links.lww.com/HC9/B889. Statistical significance was set at a 2-tailed α value of 0.1. A *p* value of <0.05 (*p < 0.05, **p < 0.01, ***p < 0.001) was considered significant. Statistical analyses were performed using R version 3.5.1 and GraphPad Prism 9.0.

## RESULTS

### Gln promotes liver mass regrowth after hepatectomy

**FIGURE 1:**
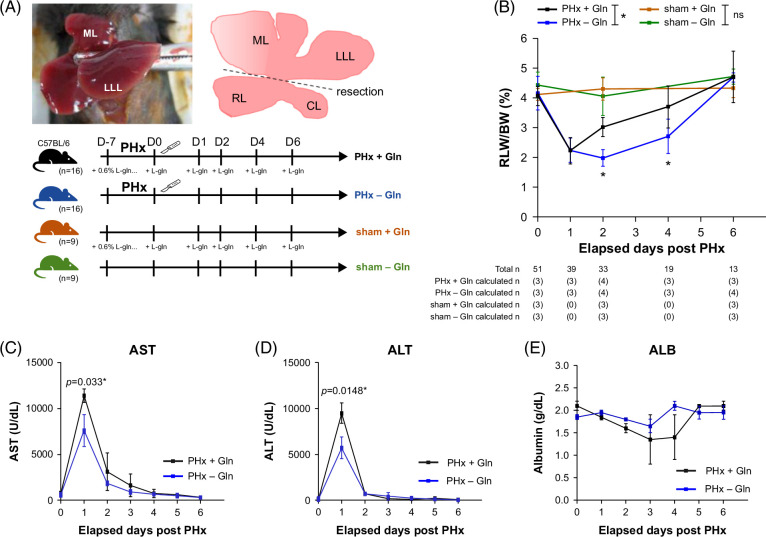
Glutamine promotes liver mass regrowth after hepatectomy. (A) Schematic diagram of the PHx murine model after resection of the ML and LLL of the liver. (B) Dynamic changes in liver mass regrowth as presented in RLW/BW according to elapsed days after PHx or sham surgery. (C) Serum AST, (D) ALT, and (E) albumin levels during the regeneration process in PHx + Gln versus PHx − Gln mice. (B) compared with mixed-effect RMs. (C–E) compared to 1-way ANOVA. ¶mixed-effect RMs *p < 0.05. Abbreviations: BW, body weight; Gln, glutamine; LLL, left lateral lobe; ML, median lobe; PHx, partial hepatectomy; RLW, residual liver weight; RMs, repeated measures.

PHx was performed in mice by resection of the median and left lateral lobes of the liver (Figure 1A). Initially, we constructed a PHx mouse model and observed that liver mass regrowth started on D2 and was completed on D6 after PHx (Supplemental Data S2 and S3, http://links.lww.com/HC9/B889). The experimental protocol is shown in the lower panel of Figure 1A. As indicated by the remnant liver weight/body weight, accelerated liver mass regrowth was observed in the PHx + Gln group compared with the PHx − Gln group, in which significant differences were noted beginning at D2 and D4 after PHx (PHx + Gln vs. PHx − Gln: 3.0 ± 0.2 vs. 2.0 ± 0.1%, *p* = 0.038 at D2; 3.7 ± 0.4 vs. 2.7 ± 0.3%, *p* = 0.020 at D4; *p* = 0.033 by mixed-effect repeated measures; Figure 1B). The mice that received sham surgery did not show significant changes in the liver mass when treated with Gln (sham + Gln vs. sham − Gln: 4.3 ± 0.2 vs. 4.1 ± 0.4%, *p* = 0.532 at D2; 4.3 ± 0.2 vs. 4.7 ± 0.1%, *p* = 0.130 at D6). Serum AST and ALT levels were significantly elevated in the PHx + Gln mice compared to those in the PHx − Gln mice after D1 and returned to a comparable baseline on D6 after PHx (PHx + Gln vs. PHx − Gln at D1: AST 11,393 ± 367 vs. 7600 ± 873 U/L, *p* = 0.033; ALT 9478 ± 564 vs. 5704 ± 588 U/L, *p* = 0.015) (Figures 1C, D). However, the serum albumin levels were comparable between the 2 groups throughout the experimental period (Figure 1E).

### Gln induces hepatocytic proliferation

**FIGURE 2:**
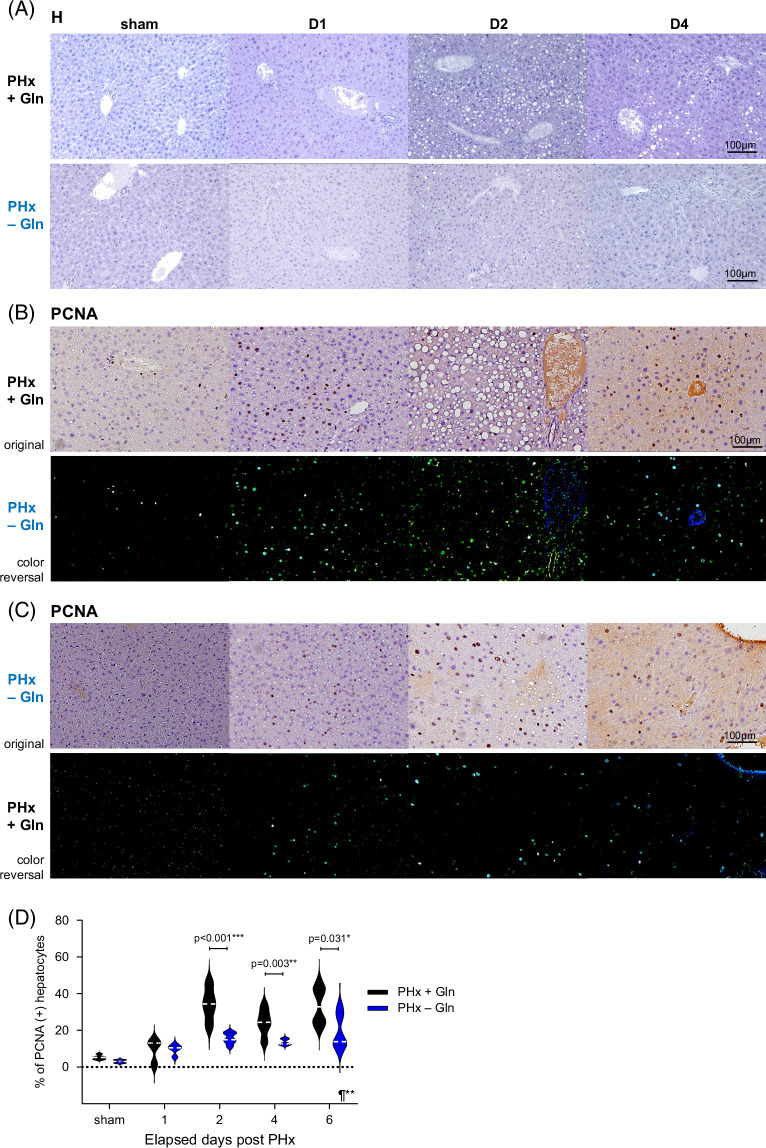
Glutamine induces hepatocytic proliferation. (A) Hematoxylin staining of liver sections from PHx + Gln versus PHx − Gln mice. (B) PCNA staining of hepatocyte nuclei in the PHx + Gln mice. (C) PCNA staining of hepatocyte nuclei in the PHx − Gln mice. (D) Violin plot for PCNA (+) hepatocytes in PHx + Gln versus PHx − Gln mice according to elapsed days after PHx. (A–C) ×200. Positive nuclei are labeled bright blue by a color reversal process enhancement (lower panel). (C) Quantification by whole-slide TissueFAXS and comparison by mixed-effect RMs and 1-way ANOVA. ¶mixed-effect RMs *p < 0.05 **p < 0.01 ***p < 0.001. Abbreviations: Gln, glutamine; H, hematoxylin; PCNA, proliferating cell nuclear antigen; PHx, partial hepatectomy; RMs, repeated measures.

Murine liver sections showed substantial morphological steatosis and parenchymal cellular disorganization from D2 to D6 in the PHx + Gln group compared with the PHx − Gln group (Figure 2A) (all results from D0 [sham] to D6 in Supplemental Data S4, http://links.lww.com/HC9/B889). Hepatocytic proliferation was determined by the density of proliferating cell nuclear antigen staining in the nuclei, and the results indicated that proliferating cell nuclear antigen positivity was greater from D1 to D4 in PHx + Gln mice (Figure 2B) than in PHx − Gln mice (Figure 2C) (PHx + Gln vs. PHx − Gln: 9.6 ± 1.5 vs. 9.1± 3.9%, *p* = 0.151 at D1; 34.3 ± 4.8 vs. 15.5 ± 1.4%, *p* < 0.001 at D2; 24.2 ± 3.3 vs. 13.6 ± 0.8%, *p* = 0.003 at D4; Figure 2D). The results demonstrated that Gln supplementation was correlated with parenchymal organization and hepatocytic proliferation after hepatectomy.

### Cell cycle, amino acid metabolism, and lipid synthesis pathways are predominantly activated in the regeneration process

**FIGURE 3:**
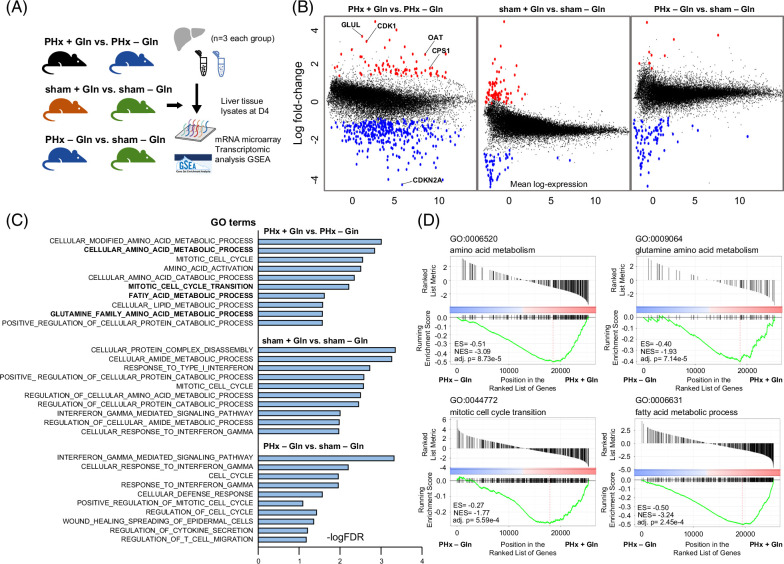
Cell cycle, amino acid metabolism, and lipid synthesis pathways are predominantly activated in the regeneration process. (A) Schematic diagram of the conditional pairs for the mRNA expression microarray. (B) Volcano plots of differentially expressed genes with dots indicating genes at significant fold-change levels in PHx + Gln versus PHx − Gln, sham + Gln versus sham − Gln, and PHx − Gln versus sham − Gln mice (red, upregulated; blue, downregulated; and black, nonsignificant) (left to right panel). Several significant candidate genes were also identified. (C) FDR levels and GO terms of the differentially expressed gene sets annotated using MSigDB murine gene sets (M5 curated gene sets). (D) Barcode plots of representative amino acid metabolism (GO:0006520), Gln metabolism (GO:0009064), mitotic cell cycle transition (GO:0044772), and fatty acid metabolism (GO:0006631) gene sets in PHx + Gln versus PHx − Gln mice by GSEA. Abbreviations: adj, adjusted; ES, enrichment score; FDR, false discovery rate; Gln, glutamine; GO, gene ontology; GSEA, gene set enrichment analysis; NES, normalized enrichment score; PHx, partial hepatectomy.

To elucidate the pathways involved and the genes differentially expressed upon hepatectomy and Glu supplementation, we conducted an mRNA expression microarray with 3 conditional pairs, namely, (1) PHx + Gln versus PHx − Gln, (2) sham + Gln versus sham − Gln, and (3) PHx − Gln versus sham − Gln, at D4, according to the experimental protocol (Figure 3A). The workflow, data processing, normalization, and quality assessment are provided in Supplemental Data S5, http://links.lww.com/HC9/B889. A total of 1228, 304, and 98 genes were significantly differentially expressed in the PHx + Gln versus PHx − Gln, sham + Gln versus sham − Gln, and PHx − Gln versus sham − Gln groups, respectively (Figure 3B). Detailed differentially expressed genes are shown in Supplemental Data S6, http://links.lww.com/HC9/B889, with several putative candidate genes highlighted. Next, we queried MSigDB for gene ontology annotations (C5) under these conditions.^29^,^30^ We observed that gene sets related to cell cycle regulation, amino acid metabolism, adipogenesis, Gln metabolism, and interferon-γ signaling were significantly altered after hepatectomy and/or glutamine supplementation (Figure 3C and Supplemental Data S7, http://links.lww.com/HC9/B889). In addition, gene set enrichment analysis barcode plots confirmed that mitotic cell cycle transition, amino acid/Gln metabolism, and fatty acid metabolism genes were more highly enriched in the PHx + Gln group than in the PHx − Gln group (Figure 3D). The results provide transcriptomic evidence of the genes and pathways involved in the response to liver mass loss and Gln supplementation.

### Gln-metabolizing enzymes and cell cycle regulation genes are expressed and correspond to the regeneration process

**FIGURE 4:**
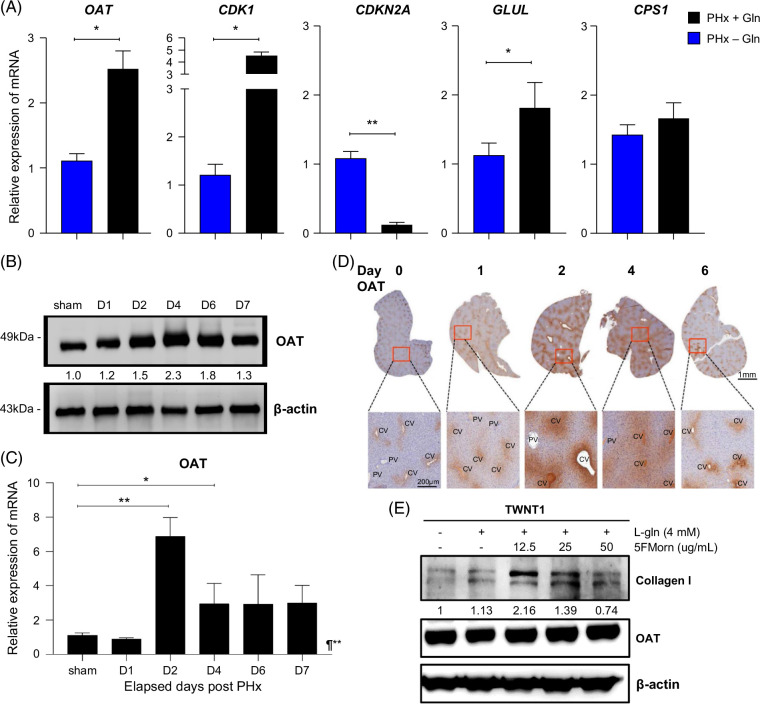
Glutamine-metabolizing enzymes and cell cycle regulation genes are expressed and correspond to the regeneration process. (A) qPCR of the mRNA levels of candidate genes in hepatocytes, such as *OAT*, *CDK1*, *CDKN2A*, *GLUL*, and *CPS1*. The relative mRNA levels were compared between PHx + Gln and PHx − Gln. (B) Immunoblotting for OAT protein expression according to the number of days after PHx in the PHx + Gln mice. (C) Relative mRNA levels of *OAT* gene expression according to elapsed days after PHx in PHx + Gln mice. (D) Spatial distribution of OAT in the liver parenchyma by IHC and highlighted by PT or CV regions according to the elapsed days after PHx in the PHx + Gln mice. (E) Immunoblotting for collagen I synthesis in TWNT-1 cells treated with l-Gln and an OAT inhibitor, 5FMorn. (A) Levels compared to PHx + Gln versus PHx − Gln with an internal reference to *ACTB* (B, E) quantified with β-actin as a reference. (C) Levels compared to those in the sham group (D0). *p < 0.05 **p < 0.01 ¶mixed-effect RMs. Abbreviations: 5FMorn, 5-fluoromethylornithine; CV, central vein; Gln, glutamine; IHC, immunohistochemistry; PHx, partial hepatectomy; PT, portal tract; qPCR, quantitative polymerase chain reaction.

To validate the expression of the putative candidate genes involved in liver regeneration after PHx, we assessed the mRNA levels in liver tissue lysates through real-time qPCR at D4. The results confirmed that the mRNA expression of ornithine aminotransferase (*OAT*) (*p* = 0.010), cyclin-dependent kinase 1 (*CDK1*) (*p* = 0.001), glutamate-ammonia ligase (*GLUL*) (*p* = 0.017), and carbamoyl-phosphate synthase 1 (*CPS1*) (*p* = 0.430) were upregulated in PHx + Gln versus PHx − Gln mice (Figure 4A). In contrast, cyclin-dependent kinase inhibitor 2A (*CDKN2A*) (*p* < 0.001) was downregulated in the PHx + Gln versus PHx − Gln group (Figure 4A). These results implied that Gln supplementation potentiated the expression of intercellular Gln cycle and cell cycle–related genes after PHx, consistent with the observations found in the mRNA expression microarray. Next, we validated the protein expression of OAT during the regeneration process through immunoblotting and found a corresponding enrichment following the regeneration timeframe, which peaked at D4 (Figure 4B). The mRNA levels also showed a compatible result, which was prominently upregulated at D2 and D4 (*p* = 0.002 compared to sham) (Figure 4C).

OAT is known to transform ornithine into glutamate (Glu), which is a key enzyme involved in the synthesis of Glu/Gln and the ammonia balance in the urea cycle, in perivenous hepatocytes.^10^,^31^,^32^ IHC for OAT in whole-slide liver sections revealed the increased expression in the PHx + Gln mice during the regeneration process (Supplemental Data S8, http://links.lww.com/HC9/B889) and was predominantly enriched in the CV instead of PT areas in the hepatic acinus, consistent with the results from the literature (Figure 4D).^31^ Next, we ought to assess the correlation of OAT activity regarding collagen synthesis upon Gln supplementation. Human HSCs (TWNT-1), which are known to regulate ECM collagen synthesis, were treated with L-Gln and the OAT inhibitor 5-fluoromethylornithine in vitro.^33^ The results demonstrated that OAT inhibition did not affect its expression but partially repressed collagen I synthesis upon Gln supplementation in a dose-dependent manner (Figure 4E). Together, these results suggest that Gln metabolic enzymes and cell cycle regulatory genes are involved in the regeneration process. Hence, OAT was one of the participants potentiated by Gln and enhanced ECM collagen synthesis during the regenerative process after PHx.

### Gln correlates with extracellular collagen synthesis and hepatocytic steatosis in the regeneration process

**FIGURE 5:**
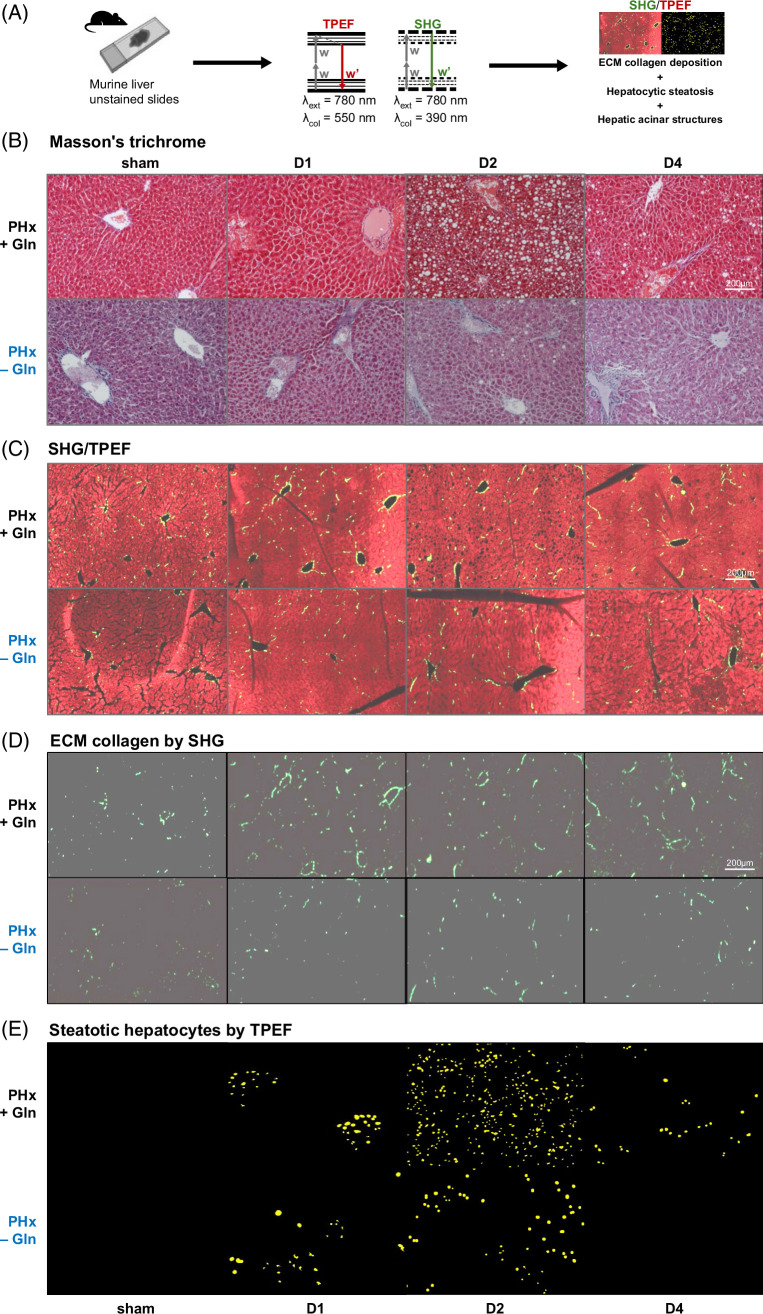
Glutamine correlates with extracellular collagen synthesis and hepatocytic steatosis in the regeneration process. (A) Illustrated diagram of algorithmic approaches in SHG/TPEF (Supplemental Data 1G). (B) Liver sections stained with Masson’s trichrome in PHx + Gln versus PHx − Gln mice. (C) SHG/TPEF merged images of liver sections from PHx + Gln and PHx − Gln mice. (D) SHG showing ECM collagen (green) in liver sections from PHx + Gln vs. PHx − Gln mice. (E) TPEF showing steatotic hepatocytes (yellow) in liver sections from PHx + Gln versus PHx − Gln mice. (B–E) ×100. (C–E) Continuous sections. Abbreviations: ECM, extracellular matrix; Gln, glutamine; PHx, partial hepatectomy; SHG/TPEF, second-harmonic generation/two-photon excitation fluorescence.

An illustration of the SHG/TPEF is shown in Figure 5A. To determine whether Gln potentiated ECM collagen deposition during the regeneration process, we first evaluated liver sections with Masson’s trichrome staining (Figure 5B) (all results from D0 [sham] to D6 in Supplemental Data S9, http://links.lww.com/HC9/B889); however, interhepatic or bridging fibrosis was barely visible on histological assessment. Alternatively, we used spatially oriented SHG/TPEF imaging for the detection and quantification of ECM collagen and hepatic steatosis. We observed significantly greater ECM collagen deposition in PHx + Gln mice than in PHx − Gln mice after PHx (Figures 5C, D). In addition, hepatocytic steatosis was more prominent in PHx + Gln mice than in PHx − Gln mice after PHx, following a similar trend (Figure 5E).

**FIGURE 6:**
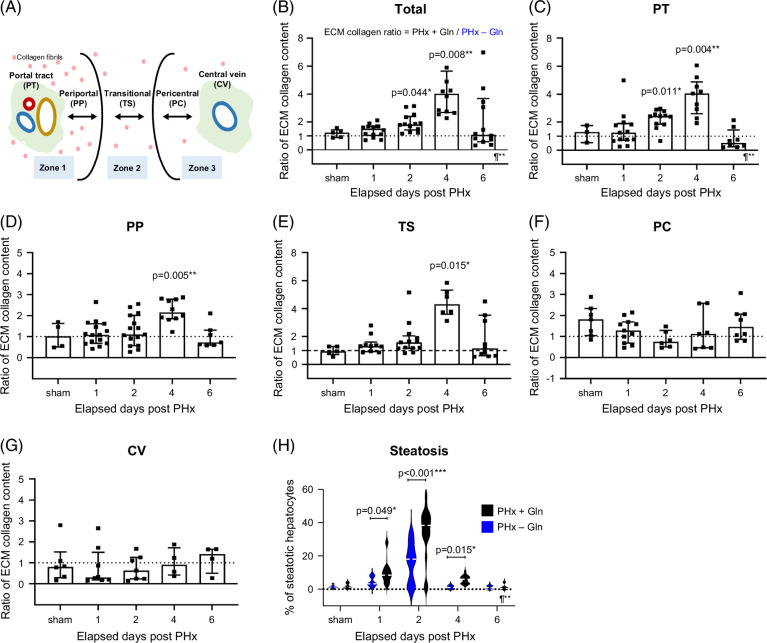
The spatial orientation of extracellular collagen and hepatocytic steatosis in the regeneration process. (A) Illustrated diagram of functional zonation within the hepatic acinus defined by TPEF. (B) Ratio of total ECM collagen from liver sections in PHx + Gln versus PHx − Gln mice treated with SHG/TPEF. Ratio of ECM collagen in (C) PT, (D) PP, (E) TS, (F) PC, and (G) CV areas from liver sections in PHx + Gln versus PHx − Gln mice by SHG/TPEF. (H) Violin plot for the percentages of steatotic hepatocytes from liver sections in PHx + Gln versus PHx − Gln mice by SHG/TPEF. (B–H) Comparison using 1-way ANOVA. *p < 0.05 **p < 0.01 ***p < 0.001 ¶mixed-effect RMs. Abbreviations: CV, central vein; ECM, extracellular matrix; Gln, glutamine; PC, pericentral; PHx, partial hepatectomy; PP, periportal; PT, portal tract; SHG/TPEF, second harmonic generation/two-photon excitation fluorescence microscopy; TS, transitional.

TPEF signals could be used to construct parenchymal structures within the hepatic acinus and merge with SHG (Figure 6A). Therefore, we observed that total ECM collagen was significantly increased at D2 and D4 by Gln supplementation (ratios in PHx + Gln / PHx − Gln: 1.2 ± 0.1, *p* = 0.044 at D2; 4.9 ± 0.9, *p* = 0.008 at D4 by paired 1-way ANOVA; Figure 6B). Excessive collagen was more prominent in PT (Figure 6C), PP (Figure 6D), and transitional (Figure 6E) areas, notably at D4, but not in pericentral (Figure 6F) or CV (Figure 6G) areas. These results revealed that the spatial predisposition for Gln potentiated ECM collagen deposition predominantly in the PT rather than in the CV area. Similarly, hepatocytic steatosis was significantly prominent at D1, D2, and D4 in the PHx + Gln mice than in the PHx − Gln mice (PHx + Gln vs. PHx − Gln: 10.3 ± 2.8 vs. 4.4 ± 1.2%, *p* = 0.049 at D1; 33.3 ± 4.0 vs. 16.2 ± 4.0%, *p* < 0.001 at D2; and 5.7 ± 0.6 vs. 1.1 ± 0.4%, *p* = 0.015 at D4; Figure 6H). These results imply that Gln is associated with ECM collagen synthesis and hepatic steatosis during the regeneration process.

### Gln accelerates structural remodeling predominantly in the PT area

**FIGURE 7:**
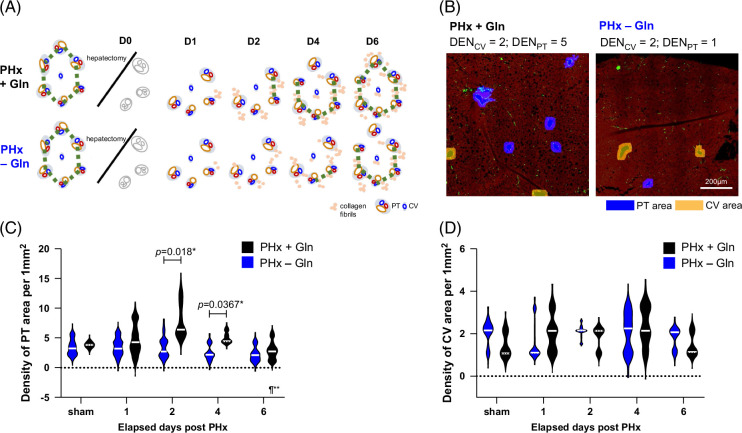
Glutamine accelerates structural remodeling predominantly in the portal tract area. (A) Schematic diagram of the structural remodeling of the PT and CV areas after PHx. (B) Representative TPEF images of the density of PT (blue) and CV (yellow) in PHx + Gln versus PHx − Gln mice. (C) Violin plot of PT density in PHx + Gln versus PHx − Gln mice by whole-slide quantification. (D) Violin plot of CV density in PHx + Gln versus PHx − Gln mice by whole-slide quantification. (B) ×100. (C, D) compared to 1-way ANOVA. *p < 0.05 ¶mixed-effect RMs. Abbreviations: CV, central vein; Gln, glutamine; PHx, partial hepatectomy; PT, portal tract; TPEF, two-photon excitation fluorescence.

In addition to ECM collagen and steatosis, we hypothesized that Gln promotes structural remodeling through the formation of PT or CV foci (Figure 7A). Liver sections were evaluated using SHG/TPEF to quantify PT and CV foci. Representative sections of the PHx + Gln and PHx − Gln mice are shown at D4 after PHx (Figure 7B), suggesting that Gln increased the density of PT (PHx + Gln vs. PHx − Gln: 5 vs. 1 per 1 mm^2^) but not CV foci (PHx + Gln vs. PHx − Gln: 2 vs. 2 per 1 mm^2^). Increased PT foci were observed in the PHx + Gln mice compared with the PHx − Gln mice from D2 to D4 (PHx + Gln vs. PHx − Gln: 7.7 ± 0.8 vs. 3.2 ± 0.5 per 1 mm^2^, *p* = 0.018 at D2; 5.0 ± 0.4 vs. 2.5 ± 0.4, *p* = 0.0367 at D4), and the difference was significant during the regeneration process (*p* < 0.01 by mixed-effect repeated measures) (Figure 7C). However, we failed to observe a difference in CV foci between the 2 groups (Figure 7D). The results demonstrated that Gln accelerated structural remodeling starting from the PT instead of the CV during liver regeneration.

## DISCUSSION

The present work revealed the effect of Gln-potentiated liver regeneration after mass loss. To the best of our knowledge, the present work is also the first study to use an AI-assisted structure-based imaging modality to describe the spatial orientation of Gln-potentiated ECM collagen synthesis and hepatic steatosis in hepatic acinus. Ito and﻿ Higashiguchi^17^ reported that i.v. Gln treatment potentiated Gln and alanine uptake and promoted hepatocytic proliferation after PHx. Kurokawa et al^34^ reported similar findings, showing that Gln plus arginine treatment increased liver mass regrowth after PHx. Hagikawa et al^35^ further reported that defective liver regeneration was partially rescued by a combination of amino acid supplementation. Although these studies showed results consistent with our present study, the spatial orientation and pathways involved were not discussed. Hepatic zonation and reorganization after mass loss correlate with complex cellular plasticity and metabolic rewiring. Using both hepatectomy and Gln as inducers for liver regeneration, our results provide insight into this well-orchestrated process with the help of a novel imaging assessment.

Rodent PHx is the most reliable and replicable model for inducing instant and substantial hepatic mass loss, which directly mimics the clinical conditions in humans. Compensatory proliferation is initiated on day 1, peaks on day 3, and recovers to the baseline at 7–15 days after resection, which finely recapitulates the priming, proliferation, and termination of liver regeneration in humans.^9^,^36^ Our results demonstrated that the liver reconstitution timeframe coincided with that in the literature. Notably, we observed a significant increase in transaminase levels in mice that received Gln supplementation during the priming period. Transamination is a critical step in Glu/Gln metabolism in the liver and can occur as a compensatory response to exogenous supply.^32^,^37^ Cooper et al^38^ described the instant uptake of ^13^N-labeled Glu into hepatocytes within seconds, which underpinned the importance of transamination reactions. Magalhães et al^18^ also reported elevations in AST and ALT but preserved bilirubin and albumin levels when hepatectomized mice were treated with Gln, which is consistent with our observations. In addition to the release of transaminases, we observed prominent hepatocytic steatosis. Walldorf et al^39^. proposed that hepatectomy induces cellular stress and thus releases fatty acids from adipose tissue to hepatocytes as an energy source for regeneration. Additional reports have indicated that steatosis after hepatectomy reflects a metabolic shift and does not impair the capacity for regeneration.^40^,^41^ Interestingly, transient versus pre-existing hepatic steatosis may contribute to a distinctive fate of liver regeneration and could be regulated by either acute or persistent endoplasmic reticulum stress.^42^ Indeed, the extensive metabolic alterations induced by hepatic mass loss may reprogram the coordinated process of regeneration.

The intercellular Gln cycle constitutes a spatially oriented regulation of Gln homeostasis in the liver. Gln is hydrolyzed by glutaminase in PP hepatocytes and participates in the urea cycle as an amino moiety donor, while perivenous hepatocytes generate and release Gln via Gln synthetase.^12^,^43^ When abundant exogenous Gln is provided, PP hepatocytes are predominantly exposed, thus accelerating the uptake of Gln. Subsequently, perivenous hepatocytes induce the high-affinity ammonia scavenger OAT to eliminate excessive ammonia through Gln synthesis.^15^,^31^ Our results indicate that Gln-metabolizing enzymes, such as OAT, GLUL (or Gln synthetase), and CPS1, and cell cycle regulatory proteins, such as the CDK family and the cell cycle checkpoint CDKN2A, were differentially expressed during the regeneration process.

In addition to metabolic reprogramming, ECM collagen deposition also plays a pivotal role in liver regeneration. Klaas et al^19^ described ECM alterations during liver injury and the abundance of collagen in the sinusoidal space of Disse. Although this study was based on toxin-induced liver injury rather than PHx, collagen was still enriched in the PT in contrast to the CV area, consistent with our observations. ECM collagen deposition, which is synonymous with hepatic fibrosis, is a fine-tuned process in a spatially oriented manner.^44^,^45^ Interestingly, these subtle alterations cannot be quantified by conventional histochemical staining (eg, Masson’s trichrome or picrosirius red) and might rely on a more sensitive imaging modality for detection, such as SHG/TPEF.

In the current study, SHG/TPEF revealed that Gln potentiated ECM collagen deposition and remodeling in a spatially oriented manner. These results highlight the relevance of PT (zone 1) instead of CV (zone 3) as the basis for liver regeneration owing to its high cellular plasticity and trans-differentiation.^46^,^47^ To date, advances in SHG/TPEF acquisition have been widely recognized and adopted for the clinical grading and quantification of hepatic fibrosis, liver cirrhosis, and NASH (qFibrosis); however, this method has not been assessed or applied elsewhere in liver regeneration.^23^,^24^,^48^ Our results may help elucidate the spatial orientation of liver regeneration and expand the utility of the laser-excited imaging quantification tool in a broader spectrum.

To the best of our knowledge, the present study is the first to use SHG/TPEF to dissect structural variations during liver regeneration. In addition, ECM collagen deposition and steatosis were observed. Furthermore, Gln supplementation has substantial clinical utility, such as in patients who plan to undergo extended hepatectomy or as healthy living liver donors. However, this study had some limitations. First, we presented the dynamics of liver regeneration in a collective manner, but not at single-cell resolution; thus, single-cell RNA sequencing might provide a deeper view. However, single-cell RNA sequencing did not contain the spatial clues described in our work. Although single-cell spatial transcriptomic analysis could address this issue, it only represents the cellular compartments and loses information on ECM. Resident cells other than hepatocytes in the microenvironment, such as HSCs, immune cells, or stromal cells, were not extensively examined in this study. Our study aimed to show the spatial orientation of liver generation potentiated by Gln and hepatectomy, and we expect the observations described herein to be applicable to other types of hepatic injuries or treatments.

## CONCLUSIONS

Gln promotes liver regeneration through cellular proliferation and metabolic reprogramming following mass loss. In contrast to the CV area, ECM collagen deposition was enriched in the vicinity of the PT and potentiated hepatic acinus remodeling during the regenerative process. Taken together, these results highlight the spatial orientation of Gln-potentiated liver regeneration and provide a potential research rationale for clinical studies.

## Supplementary Material
