# Supplementary material for: Second harmonic generation microscopy reveals the spatial orientation of glutamine-potentiated liver regeneration after hepatectomy
Source: Hepatol Commun. 2025 Mar 7;9(3):e0640. doi: 10.1097/HC9.0000000000000640 (PMC11888978; doi:10.1097/HC9.0000000000000640)
Supplement: Supplementary file 1 [file hc9-9-e0640-s001.pdf]

Supporting information

Second Harmonic Generation Microscopy Reveals the Spatial Orientation of Glutamine-potentiated Liver Regeneration After Hepatectomy

Chia-Jui Yen, MD., PhD.

Department of Oncology, National Cheng Kung University Hospital, College of Medicine, National Cheng Kung University, Tainan, Taiwan

yencj@mail.ncku.edu.tw

Supporting Data 1 2

Supporting Data 2 8

Supporting Data 3 9

Supporting Data 4 10

Supporting Data 5 11

Supporting Data 6 15

Supporting Data 7 24

Supporting Data 8 29

Supporting Data 9 30

Supplemental Data 1. supporting information on materials and methods

A. Partial hepatectomy murine model

Eight-week-old C57BL/6 mice were anesthetized with intraperitoneal injection of 15 mg/kg of Zoletil 50 and 5 mg/kg of Rompun. After anesthesia, 70% partial hepatectomy (PHx) was conducted with a midline laparotomy, a resection of the median and left lateral lobes (ML + LLL), and followed by remnant stump ligation for hemostasis, as shown in Figure 1A.[1] Mice in the sham group received a midline laparotomy in the absence of hepatic resection and followed by identical procedures as the PHx group (Supplemental Data 2). The remnant liver weight/body weight results for mice receiving PHx are shown in Supplemental Data 3. L-glutamic acid 5-amide (Gln) was obtained from MilliporeSigma, MA, US and mice were fed with 0.6% Gln versus PBS in the drinking water starting at 7 days prior to PHx and another 6 days after Phx. The mice were randomized into four groups, which were 1) sham with Gln supplementation (sham + Gln), 2) sham without Gln supplementation (sham – Gln), 3) PHx with Gln supplementation (PHx + Gln) and 4) PHx without Gln supplementation (PHx – Gln). Remnant livers were collected for further analyses and weighted daily from Day 1 to 6 post PHx. The percentages of the remnant liver weight by body weight (RLW/BW) were calculated to compare the dynamics of liver mass regrowth.

B. Immunohistochemistry

The slides were incubated with rabbit anti-proliferating cell nuclear antigen (PCNA) antibody (Clone 100539; GeneTex, CA, US) and anti-ornithine aminotransferase (OAT) antibody (sc-374243; Santa Cruz, CA, US)) at 1:100 to 1:500 dilution overnight at 4 °C, and then incubated sequentially with biotin-conjugated secondary antibody (DAKO, Denmark) and streptavidin-horseradish peroxidase

(DAKO). Subsequently, the peroxidase activity was detected by using a Bond Polymer Refine Detection Kit (Leica Biosystem, Germany) and counterstaining was performed with hematoxylin (DAKO). Masson's trichrome staining (HT-15 Masson kit; MilliporeSigma, MA, US) was used to visual the fibrosis in the liver parenchyma. Briefly, the nucleus and cytoplasm were stained by using Weigert's iron hematoxylin with black color and Biebrich scarlet-acid fuchsin with red color, respectively. Collagen was stained in blue color by phosphotungstic and phosphomolybdic acid. All IHC or special stained sections were photographed by using an optical microscope (Olympus BX61; Tokyo, Japan) and analyzed by TissueFAXS (TissueGnostics, Austria).

C. Immunoblotting

Total tissue lysates from liver samples and the protein concentration of the supernatants were measured using the amido-black method. Equal amounts of proteins and 4-fold loading dye was prepared and heated at 100 °C for 10 minutes. Protein samples were separated by 8%, 10%, 12% or 15% SDS-PAGE and transferred to PVDF membrane (MilliporeSigma) at 120 volt for 1.5 to 2.0 hours. Immuno-detection was performed using antibody against OAT (sc-374243; Santa Cruz, CA, US) at 1:1000 dilution. Protein expression was visualized by SuperSignal West Pico Chemiluminescent Substrate (Thermo Fisher Scientific) and quantified by a comparison with β-actin.

D. TissueFAXS imaging quantification

Sample slides were prepared on formalin-fixed paraffin-embedded sections and stained with IHC. Images were captured from the TissueFAXS platform (TissueGnostics, Austria) and analyzed by TissueQuest software (TissueGnostics, Austria) in the Core laboratory, Center of Clinical Medicine,

National Cheng Kung University Hospital, Tainan, Taiwan. Whole-slide images were recorded with a digital camera (DC500; Leica Biosystem, Germany) on an inverted fluorescence microscope (Olympus BX61). Images were captured from the TissueFAXS platform (TissueGnostics, Austria) and analyzed by TissueQuest software (TissueGnostics, Austria) in the Core laboratory, Center of Clinical Medicine, National Cheng Kung University Hospital, Tainan, Taiwan. For quantifications of positive stained cells, ten to fifteen selected regions of interests (ROIs) by 1 mm x 1 mm were computed and taken as an average. Nuclei of the stained cells were detected by dissection algorithms in the DAPI channel. Positive cells were computed by either non-annular signal grow around the nuclei or gated staining intensity within the prespecified area. All positive signals were plotted against DAPI signals in flow cytometry-like 2D scattergrams.

E. Gene expression microarray and gene set enrichment analysis

The quality of mRNA samples was confirmed by Bioanalyzer 2100 (Agilent) with RNA 6000 Nano LabChip (Agilent). A pooling result of mRNA expression levels from sham group was used as reference. RNA amplification, labeling, hybridization and washing procedures were conducted as described by Kuo et al.[3] Selected genes with distinctive expression levels were analyzed using the significance analysis of microarray (SAM) procedure with a false discovery rate (FDR) of 0.05. R version 4.3.0 was used to analyze the processed data with standard protocols and algorithm by package *edgeR*, *Limma* and *Glimma*. Gene set enrichment analysis (GSEA) was conducted with *MSigDB* using the M2 curated murine gene sets.[4] Statistically significant gene sets were compared according to the FDR.

F. Second harmonic generation/two-photon excitation fluorescence microscopy image acquisition

Second harmonic generation /two-photon excitation fluorescence microscopy (SHG/TPEF) was used in the study to elucidate the spatial information on extracellular materials in liver parenchyma.[9] SHG imaging was able to capture extracellular material deposition, while TPEF microscopy identified the associated spatial architecture. The signals were laser-excited at 780 nm and SHG recorded at 390 nm while TPEF at 550 nm, respectively.

Using Otsu's automatic threshold method, the location of liver tissue and collagen was detected from the TPEF channel and the SHG channel, respectively.[10] The lumens inside the liver tissue, including blood vessels, bile ducts, fat vacuoles and tissue cracks, can be identified based on the morphological characteristics, such as the density, width and length of lumens, and the collagen features surrounding the lumens. The blood vessels and bile ducts were distinguished from others by a decision tree, which was constructed by classification and regression tree (CART) model. Then, multiple structures were constructed based on the distances among the lumens and the surrounding collagen of these holes. Each structure represented a portal tract (PT) region or a central vein (CV) region, which can be recognized by another CART with features such as the number of lumens, the total area of lumens, the area of maximal lumens, and the area of collagen. After identifying the PT and CV region, the Periportal (PP) and pericentral (PC) can be determined. The PP is the region within 50 μm around the portal tract while PC is the region within 50 μm around the central vein. Transitional (TS) is the remaining region excluding PT, CV, PP and PC from the overall tissue, as shown in below.

With SHG signal acquisition, a total of 87 collagen architectural features were obtained. Principal component analysis (PCA) with multinominal regression was applied to reduce the dimensions of the features for additional computation. In the current study, liver specimens were proceeded for image acquisition using SHG/TPEF (Genesis system; HistoIndex Pte., Singapore).[5,6]

G. Special statistical considerations for pairwise comparisons

In conditions where pairwise multiple comparisons were made, the results were adjusted according to Benjamini-Hochberg (BH) method as presented by false discovery rates.[7] In the time-dependent repeated measures (RMs) of variables, a mixed-effect model analysis was used to compare between group differences (PHx + Gln vs. PHx – Gln).[8] Crude discrete data was standardized by means and standard deviations of the group to eliminate the huge heterogeneity among conditions. Outlier estimates were removed if the data points fall over the range of 2.5 standard deviation away from the crude mean.

References

1. Mitchell C, Willenbring H. A reproducible and well-tolerated method for 2/3 partial hepatectomy in mice. Nature protocols 2008;3:1167-70.
2. Lohnes K, Quebbemann NR, Liu K, Kobzeff F, Loo JA, Ogorzalek Loo RR. Combining high-throughput MALDI-TOF mass spectrometry and isoelectric focusing gel electrophoresis for virtual 2D gel-based proteomics. Methods (San Diego, Calif) 2016;104:163-9.
3. Kuo JH, Jan MS, Lin YL, Lin C. Interactions between octaarginine and U-937 human macrophages: global gene expression profiling, superoxide anion content, and cytokine production. Journal of controlled release : official journal of the Controlled Release Society 2009;139:197-204.
4. Castanza AS, Recla JM, Eby D, Thorvaldsdóttir H, Bult CJ, Mesirov JP. Extending support for mouse data in the Molecular Signatures Database (MSigDB). Nature Methods 2023;20:1619-20.
5. Zoumi A, Yeh A, Tromberg BJ. Imaging cells and extracellular matrix in vivo by using second-harmonic generation and two-photon excited fluorescence. Proceedings of the National Academy of Sciences 2002;99:11014-9.
6. Wang Y, Wong GL, He FP, et al. Quantifying and monitoring fibrosis in non-alcoholic fatty liver disease using dual-photon microscopy. Gut 2020;69:1116-26.

7. Benjamini Y, Hochberg Y. Controlling the False Discovery Rate: A Practical and Powerful Approach to Multiple Testing. Journal of the Royal Statistical Society Series B (Methodological) 1995;57:289-300.
8. Detry MA, Ma Y. Analyzing Repeated Measurements Using Mixed Models. JAMA 2016;315:407-8.

9. Zoumi, A., Yeh, A. & Tromberg, B. J. Imaging cells and extracellular matrix in vivo by using second-harmonic generation and two-photon excited fluorescence. Proceedings of the National Academy of Sciences 99, 11014-11019, doi:10.1073/pnas.172368799 (2002).

10. Barros WKP, Dias LA, Fernandes MAC. Fully Parallel Implementation of Otsu Automatic Image Thresholding Algorithm on FPGA. Sensors. 2021; 21(12):4151. https://doi.org/10.3390/s21124151

Supplemental Data 2: the murine partial hepatectomy model

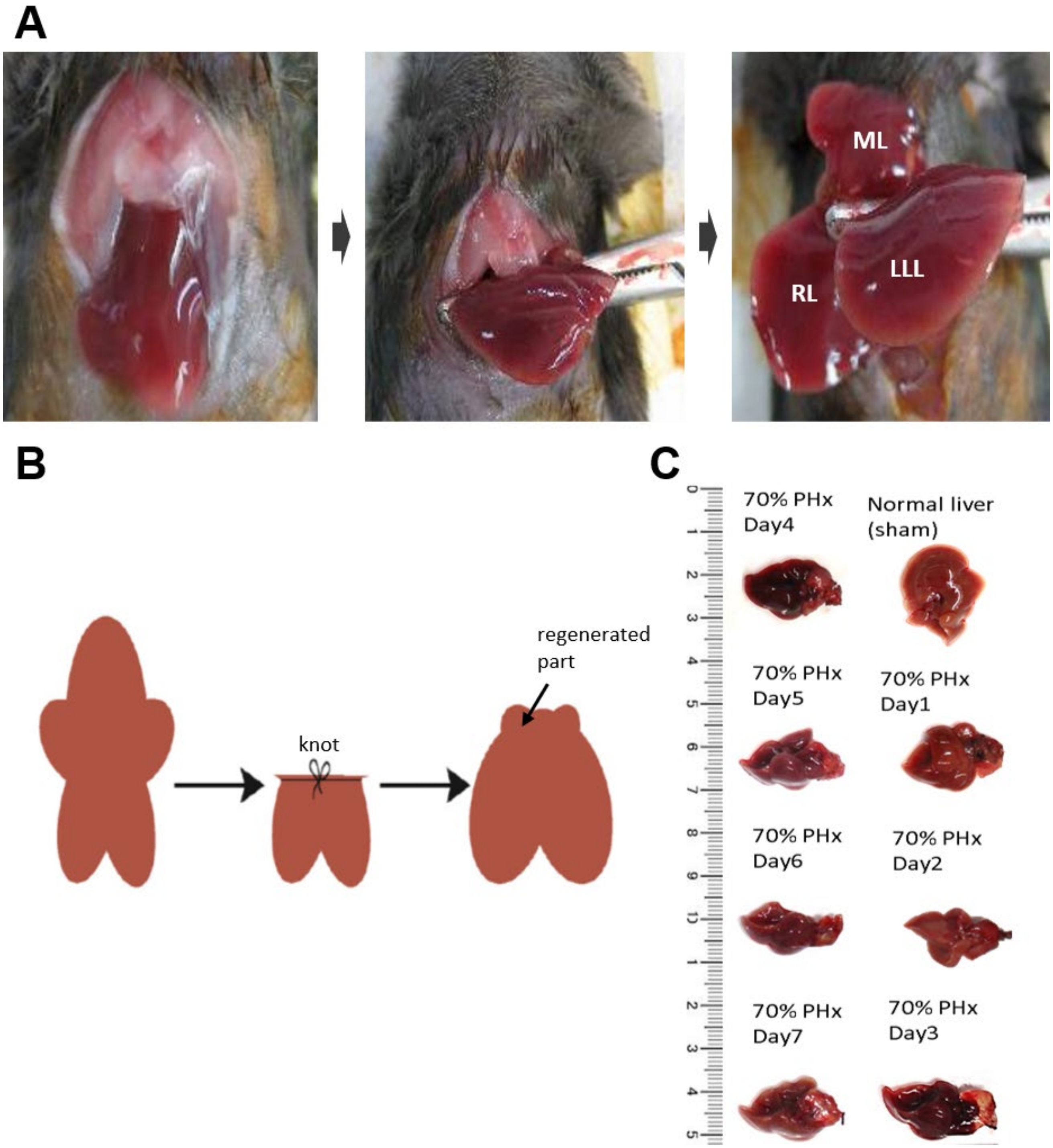

(A) Procedures of murine PHx. The ML and LLL of the liver were resected upon a surgical knot ligation. (B) After proper ligation and knotting, the regenerated part is expected to appear at the knot area. (C) The appearance of the remnant liver according to the elapsed days post PHx.

Supplemental Data 3: dynamic changes of remnant liver versus body weight

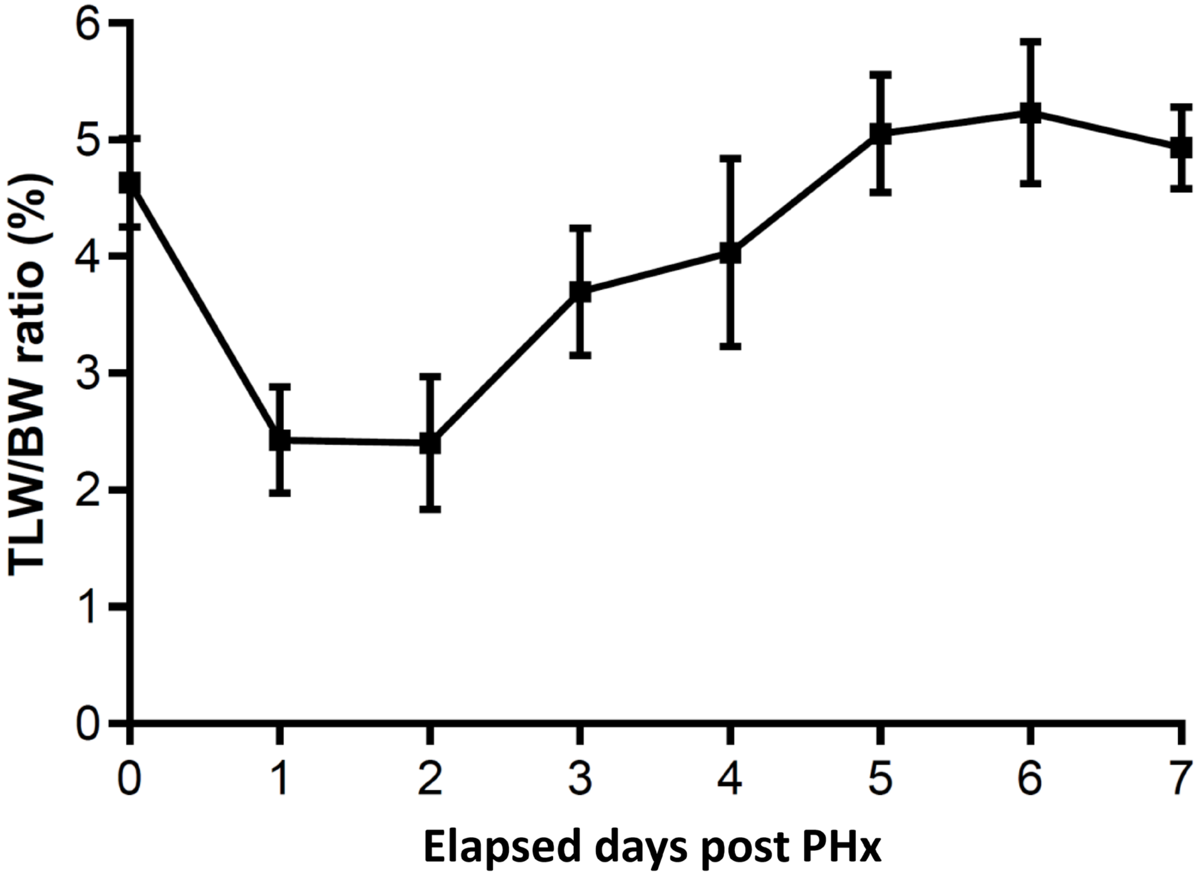

Total n	28	25	21	17	14	10	7	3
Calculated n	(3)	(4)	(4)	(3)	(4)	(3)	(4)	(3)

The dynamic changes of liver mass regrowth after 70% PHx as presented in RLW/BW (%). Total n was presented based on the number of mice at the specific time point post PHx. Calculated n was presented based on the number of mice which were sacrificed for calculating the RLW/BW at the specific time point post PHx.

RLW/BW, residual liver weight/body weight; PHx, partial hepatectomy; Total n, total number; Calculated n, calculated number.

Supplemental Data 4: PCNA staining of liver sections from D0 (sham)-D6

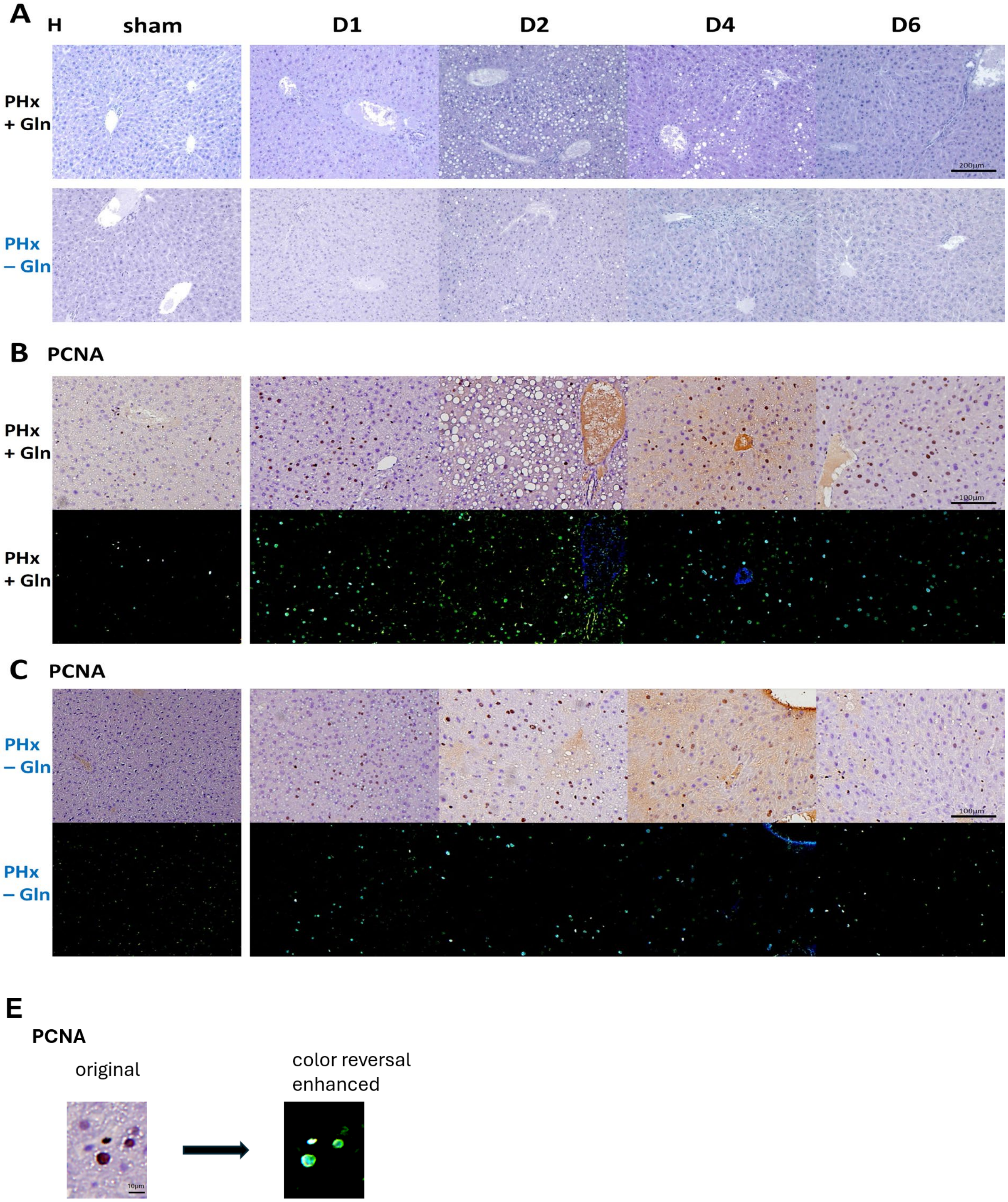

(A) Hematoxylin staining of liver sections from PHx + Gln versus PHx – Gln mice. (B) PCNA staining of hepatocyte nuclei in the PHx + Gln mice. (C) PCNA staining of hepatocyte nuclei in the PHx-Gln mice. (D) Violin plot for PCNA (+) hepatocytes in PHx + Gln vs. PHx – Gln mice according to elapsed days post-PHx. (E) An example of color reversal of PCNA-positive nuclei.

Supplemental Data 5: workflow and quality assessment of mRNA expression microarray

1) Analytic workflow

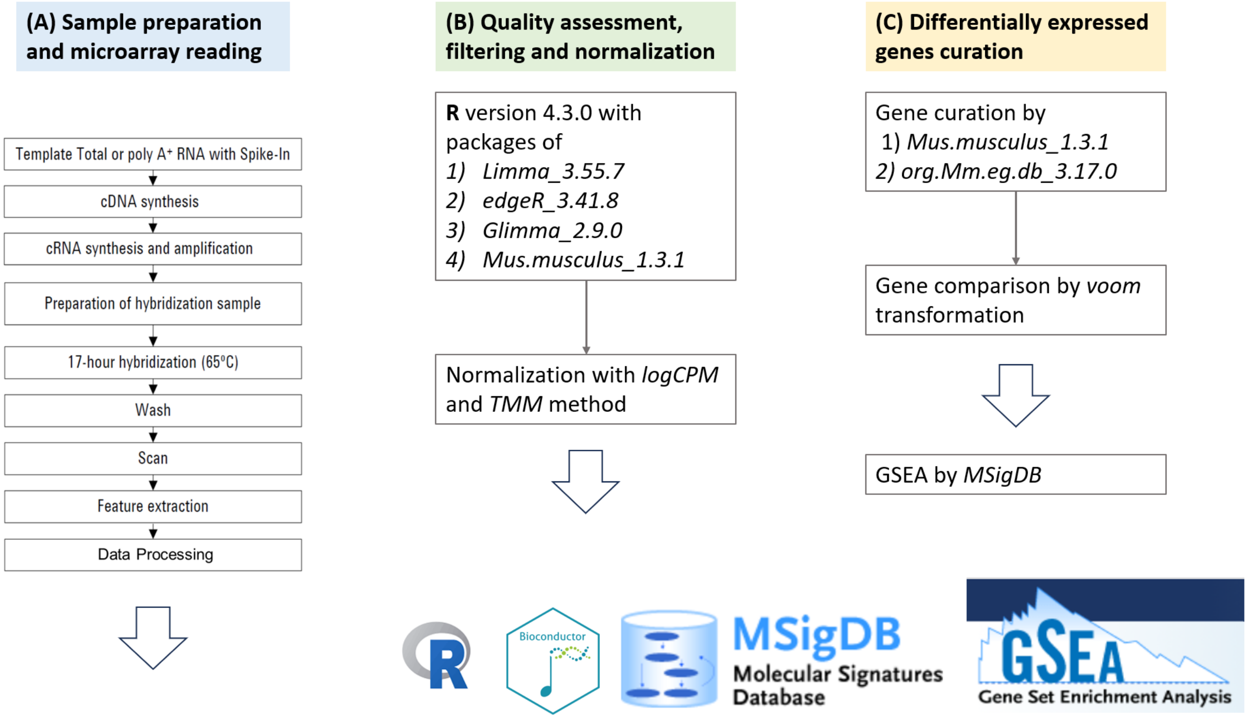

mRNA microarray procedures: 0.2 µg of total RNA was amplified by a Low Input Quick-Amp Labeling kit (Agilent Technologies, USA) and labeled with Cy3 (CyDye, Agilent Technologies, USA) during the in vitro transcription process. 0.6 µg of Cy3-labled cRNA was fragmented to an average size of about 50-100 nucleotides by incubation with fragmentation buffer at 60°C for 30 minutes. Correspondingly fragmented labeled cRNA is then pooled and hybridized to Agilent SurePrint G3 Mouse Gene Exp v2 Array Kit (Agilent Technologies, USA) at 65°C for 17 h. After washing and drying by nitrogen gun blowing, microarrays are scanned with an Agilent microarray scanner (Agilent Technologies, USA) at 535 nm for Cy3. Scanned images are analyzed by Feature extraction10.7.3.1 software (Agilent Technologies, USA), an image analysis and normalization software is used to quantify signal and background intensity for each feature.

2) mRNA quality control assessment report

	Sample name	Sample volume (ul)	RNA conc. (ng/µl)	RNA quantity (ng)	OD260/280 Ratio	Bioanalyzer chip lane location	28S/18S Ratio	RIN
#1	PHX_1	9	310.7	1242.8	2.05	1	1.1	8.9
#2	SHAM_1	9	316.2	1264.6	2.04	2	1.3	9.2
#3	PHX+2%GLN_1	9	279.3	838.0	1.94	3	1.2	9.3
#4	SHAM+2%GLN_1	9	324.8	974.5	2.06	4	1.4	9.4
#5	PHX_2	9	267.9	1071.7	2.00	5	1.3	9.3
#6	SHAM_2	9	194.3	582.8	1.93	6	1.3	9.3

#7	PHX+2%GLN_2	9	303.2	1212.6	2.08	7	1.1	8.8
#8	SHAM+2%GLN_2	9	325.8	1303.3	2.00	8	1.1	9.0
#9	PHX_3	9	328.21	1312.8	2.06	9	1.2	9.0
#10	SHAM_3	9	309.50	1238.0	2.02	10	1.2	9.5
#11	PHX+2%GLN_3	9	347.90	1391.6	2.06	11	1.3	9.3
#12	SHAM+2%GLN_3	9	396.90	1587.6	1.98	12	1.2	9.3

3) mRNA reads normalization and standardization procedures

a) *LogCPM* adjustment for heterogeneity

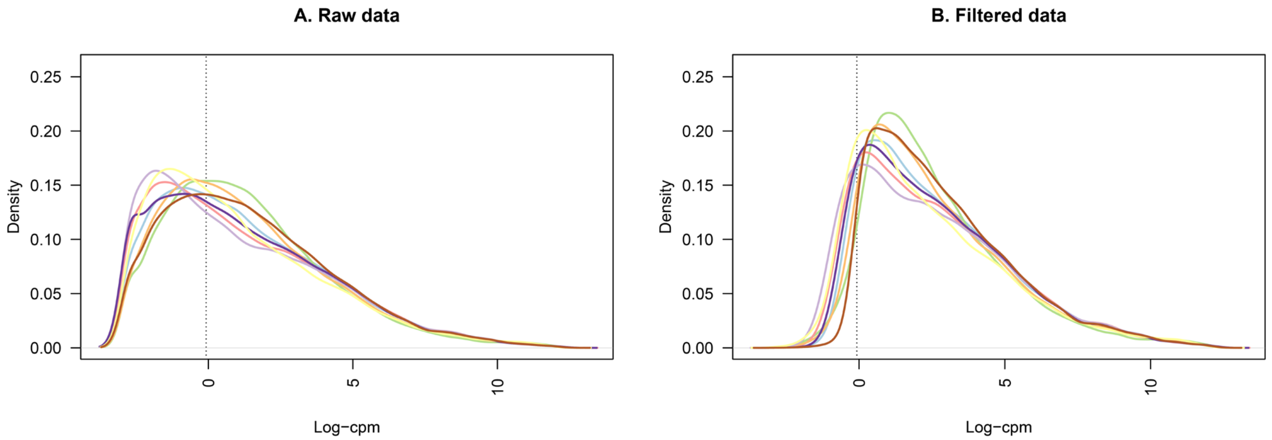

b) *TMM* for library size normalization

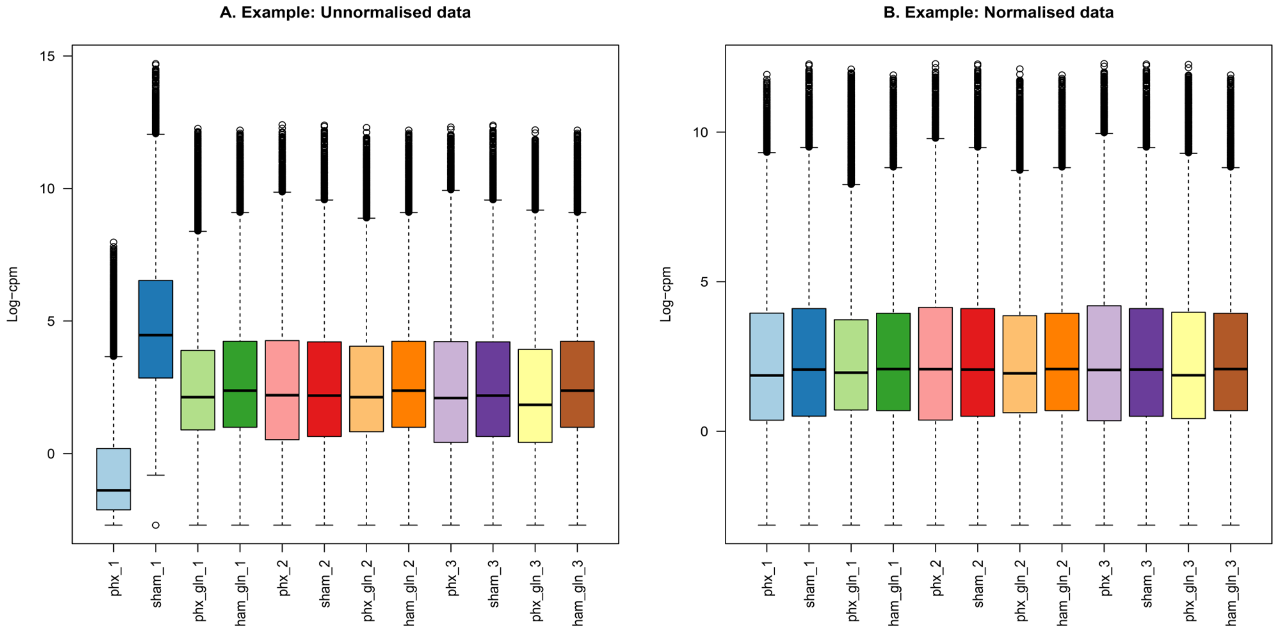

c) *Voom* mean-variance calibration

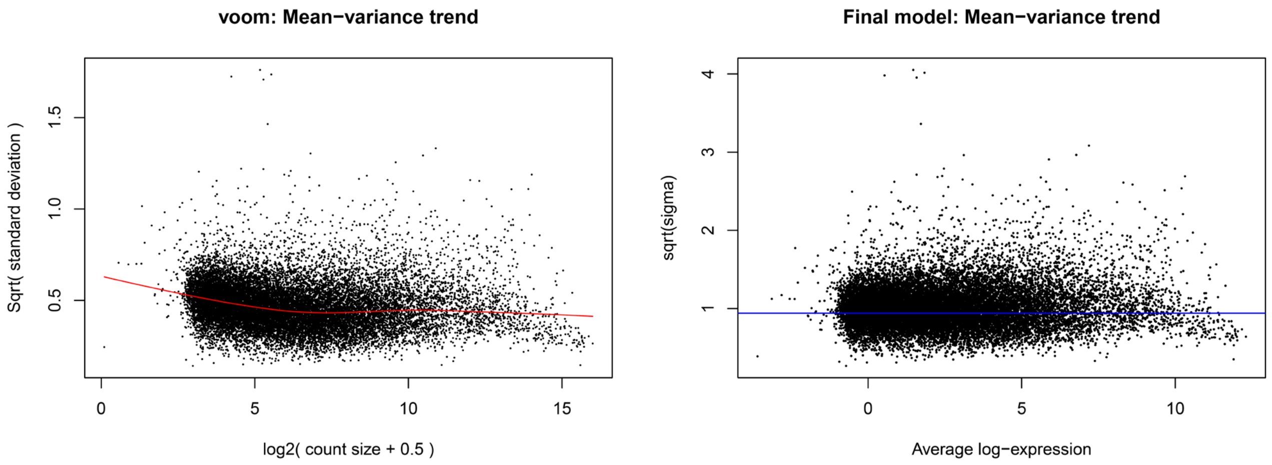

4) R session and workspace

R version 4.3.0 (2023-04-21 ucrt)

Platform: x86_64-w64-mingw32/x64 (64-bit)

Running under: Windows 10 x64 (build 19045)

Matrix products: default

locale:

LC_COLLATE=Chinese (Traditional)_Taiwan.utf8 LC_CTYPE=Chinese (Traditional)_Taiwan.utf8

LC_MONETARY=Chinese (Traditional)_Taiwan.utf8 LC_NUMERIC=C

LC_TIME=Chinese (Traditional)_Taiwan.utf8

time zone: Asia/Taipei tzcode source: internal

attached base packages:

stats4   stats   graphics grDevices utils   datasets methods   base

other attached packages:

gplots_3.1.3   Mus.musculus_1.3.1

TxDb.Mmusculus.UCSC.mm10.knownGene_3.10.0 org.Mm.eg.db_3.17.0

GO.db_3.17.0   OrganismDbi_1.42.0

GenomicFeatures_1.52.0 GenomicRanges_1.51.4

GenomeInfoDb_1.35.17 AnnotationDbi_1.61.2

IRanges_2.33.1 S4Vectors_0.37.7

Biobase_2.59.0 BiocGenerics_0.45.3

Glimma_2.9.0 edgeR_3.41.8

limma_3.55.7

Supplemental Data 6: differentially-expressed genes

1) Top 100 differentially-expressed genes in PHx + Gln vs. PHx – Gln

Symbol	logFC	Avg. expression	t	Crude p	FDR	-logFDR
Inhbb	3.486755	2.224049	30.89167	3.04403092462802e-18	7.55193632090965e-14	13.12194
**Oat**	2.836031	7.173841	23.34674	6.00974403334697e-16	4.96985799077683e-12	11.30366
Car3	-7.52718	2.368505	-22.3404	1.3789465558043e-15	8.55257127573724e-12	11.0679
Klk1b22	-3.6685	0.893828	-20.4368	7.18748845020148e-15	3.56628801922097e-11	10.44778
Mid1ip1	-2.32068	5.070163	-19.7584	1.34288500456485e-14	5.55260567970821e-11	10.2555
Apol9a	-4.42772	7.5861	-17.8665	8.56108514677666e-14	2.35991068229314e-10	9.627104
Esco2	-6.10845	-1.08378	-17.6936	1.02483642104186e-13	2.54251667696274e-10	9.594736
Tg	5.774553	-0.46847	17.11469	1.87963235373975e-13	4.23925446035722e-10	9.372711
Cyp2c55	-4.64778	2.824991	-16.8961	2.37219753283363e-13	4.9043207160058e-10	9.309421
Lyve1	2.304076	3.167483	16.48504	3.70946171006997e-13	7.07907965885584e-10	9.150023
Aqp8	-5.13206	6.466415	-16.3301	4.40489202286806e-13	7.80578329966669e-10	9.107584
Bdh2	-2.34403	5.009178	-15.8439	7.60175110314072e-13	1.17869901948636e-09	8.928597
Prtn3	-3.2272	3.038244	-15.7302	8.6565872937361e-13	1.26330161276646e-09	8.898493
**Cdkn2a**	-7.56559	8.315022	-15.2275	1.56439559720175e-12	2.15617168727657e-09	8.666317
Itga2	4.113134	-2.76244	14.71821	2.8536180759423e-12	3.21797322027511e-09	8.492418
Tmem14a	-3.69315	2.435953	-14.7613	2.70823732863238e-12	3.21797322027511e-09	8.492418
Cidec	6.375697	3.256014	14.4103	4.17189816449921e-12	4.50002702448091e-09	8.346785
Limd2	-3.40393	1.585864	-14.2195	5.27026794457876e-12	5.4479198932106e-09	8.263769
Map3k6	2.454376	1.396302	13.887	8.01378326610082e-12	7.64669034802674e-09	8.116526
Klk1b3	-2.6356	1.660244	-13.7117	1.00285049769195e-11	9.214710369348e-09	8.035518
Psrc1	-5.15879	-0.9894	-13.6198	1.13008395702074e-11	1.00129474606169e-08	7.999438
Hsd3b6	-6.42325	2.313455	-13.5187	1.29182912665364e-11	1.08679804049894e-08	7.963851
**Cps1**	2.0423	8.297693	13.44564	1.41612129428876e-11	1.11965779526759e-08	7.950915
Dclk3	-2.95641	2.560029	-13.4306	1.4441956325754e-11	1.11965779526759e-08	7.950915
Hsd3b3	-5.46932	3.59583	-13.3227	1.66621559102651e-11	1.25264068478111e-08	7.902173
Igfbp1	4.120942	8.651105	13.11872	2.18082146269249e-11	1.59129410788053e-08	7.79825
Ly6e	-2.79852	6.944985	-12.9989	2.56004891193902e-11	1.76422926267487e-08	7.753445
Sult1c2	-2.77771	2.831921	-13.0108	2.51935798760799e-11	1.76422926267487e-08	7.753445
Zbp1	-3.54913	0.604422	-12.9685	2.66662139173966e-11	1.78800567858565e-08	7.747631
Olfm3	-1.89852	1.54529	-12.9039	2.90947168839069e-11	1.89950218729696e-08	7.72136
Lgals1	-3.60411	5.008648	-12.744	3.61554239648357e-11	2.29994849524002e-08	7.638282
Keg1	-6.00935	4.580694	-12.6979	3.86007950418684e-11	2.39411781048429e-08	7.620854
Serpina3c	1.932147	8.564287	12.54391	4.75888136094789e-11	2.87958750448186e-08	7.54067
Cdca3	-3.96709	2.296674	-12.3529	6.20773131145148e-11	3.5815722350186e-08	7.445926

Adam11	-2.44507	2.434745	-12.2779	6.8948969273088e-11	3.718597779774e-08	7.429621
Lpl	-2.98807	2.475982	-12.2803	6.8722395533768e-11	3.718597779774e-08	7.429621
Smim22	-2.20996	5.528517	-12.283	6.84551819932629e-11	3.718597779774e-08	7.429621
Ly6c2	-2.96249	6.332547	-12.1703	8.02584215467939e-11	4.14818995865502e-08	7.382141
Tmem132a	-2.05631	-0.05619	-12.1548	8.20336593374004e-11	4.15341439694197e-08	7.381595
Serpine1	4.719555	1.985709	12.10119	8.85635352404058e-11	4.39434549155845e-08	7.357106
Cmpk2	-3.04952	1.409471	-11.9803	1.05189980223243e-10	5.1169769007028e-08	7.290987
Clec2h	-5.20395	2.882967	-11.903	1.17668336868363e-10	5.61391109493698e-08	7.250734
Srebf1	-2.7506	7.046935	-11.6369	1.73033131388924e-10	7.94959066042187e-08	7.099655
Phlda3	-2.8459	1.811709	-11.4558	2.25987711965709e-10	1.01936893566496e-07	6.991669
**Cdk1**	3.162187	2.348366	11.218	3.22418876736093e-10	1.37911895050788e-07	6.860398
Apol9b	-5.97634	7.369103	-11.122	3.73764665835963e-10	1.57164874486854e-07	6.803645
Gbp3	-3.15893	2.950844	-11.0357	4.25056858125269e-10	1.72543519606512e-07	6.763101
Olfml1	-2.43795	2.768778	-11.0263	4.31202314305444e-10	1.72543519606512e-07	6.763101
Kdm5d	-2.251	-3.35299	-11.0061	4.44656505976188e-10	1.75102908837512e-07	6.756707
Cyp2c70	-3.97458	9.169678	-10.9332	4.97229083311128e-10	1.92746192622903e-07	6.715014
**Glul**	3.19068	2.732086	10.8428	5.71475228269403e-10	2.18118906740548e-07	6.661307
Hamp2	-5.06612	5.828166	-10.7611	6.49164816330283e-10	2.44017120126333e-07	6.61258
Tmprss4	-2.49089	-0.29419	-10.7125	6.99582823204743e-10	2.59044033744574e-07	6.586626
Birc5	-3.83798	3.655924	-10.6058	8.26695066717401e-10	2.97238810292638e-07	6.526894
Lipc	-3.84738	3.037502	-10.6113	8.19646549048383e-10	2.97238810292638e-07	6.526894
Apoa4	4.08737	6.398267	10.59182	8.4510621518944e-10	2.99517715609069e-07	6.523577
Stmn1-rs1	-3.87681	4.577684	-10.5313	9.29617539629331e-10	3.24829317474142e-07	6.488345
Gck	-3.3458	3.824997	-10.4551	1.04856835008868e-09	3.46852429298001e-07	6.459855
Smim6	-3.15587	-0.38705	-10.4343	1.083761754581e-09	3.53776912755264e-07	6.451271
Cd83	-2.58053	0.41775	-10.4139	1.11949199061613e-09	3.6069450383371e-07	6.44286
Mki67	-4.57247	2.705629	-10.3738	1.19374642784246e-09	3.79687886260815e-07	6.420573
Chek2	-3.57907	-1.90873	-10.3253	1.28954117837901e-09	3.9496576659759e-07	6.403441
Sntg2	-3.89	1.612015	-10.3312	1.27741985356204e-09	3.9496576659759e-07	6.403441
Dock8	-3.35573	0.914426	-10.3147	1.31163404259115e-09	3.95217109445784e-07	6.403164
Ihh	-3.17714	1.39723	-10.3096	1.32222258390101e-09	3.95217109445784e-07	6.403164
Cenpa	-2.6293	1.924753	-10.2557	1.44179849662954e-09	4.25828320272409e-07	6.370765
Dck	-2.65503	1.418187	-10.1886	1.60640888394256e-09	4.58085034502655e-07	6.339054
Gbp2b	-3.80534	0.38283	-10.0673	1.95589455329845e-09	5.45211100817766e-07	6.263435
Ppp1r14a	-3.27101	2.093161	-10.0726	1.93897261037598e-09	5.45211100817766e-07	6.263435
Mmd2	-3.4683	2.002776	-10.0442	2.03078064203027e-09	5.59795966090322e-07	6.25197
Rasd1	2.194301	-0.52506	9.998662	2.1877309106797e-09	5.96433144648931e-07	6.224438
Hyi	-2.06517	6.352476	-9.89132	2.60967792865536e-09	6.98602772227627e-07	6.15577

Ifit3	-4.20598	3.174771	-9.88934	2.61881002124912e-09	6.98602772227627e-07	6.15577
Isg15	-3.54126	4.961548	-9.84757	2.80529174100816e-09	7.32594555817594e-07	6.135136
Slc9a9	-2.93997	2.117392	-9.84878	2.79958729383202e-09	7.32594555817594e-07	6.135136
Rnf186	-3.82874	2.98942	-9.81914	2.94067214845141e-09	7.52114797226093e-07	6.123716
Gsta4	-5.4118	3.523622	-9.76662	3.21379126508956e-09	8.05363106016231e-07	6.094008
Gfra1	1.744288	3.438286	9.746622	3.31692995985759e-09	8.22897153741069e-07	6.084654
Itprip	1.764028	2.357302	9.7171	3.48429452054141e-09	8.5553563101427e-07	6.067762
Uba7	-2.92654	3.465518	-9.71143	3.5174587594605e-09	8.5553563101427e-07	6.067762
Fancb	-2.70705	-0.17005	-9.65665	3.85506645290382e-09	9.28547025534863e-07	6.032196
Mmp15	-4.10948	0.431956	-9.50438	4.98372709448124e-09	1.18885851429793e-06	5.92487
Serpinh1	-2.2394	2.368389	-9.4659	5.31890554680194e-09	1.25673074010104e-06	5.900758
Rdm1	-2.45049	0.735949	-9.4159	5.79127662783715e-09	1.34276431644871e-06	5.872
Pcolce	-2.10727	4.198443	-9.38899	6.06339225726343e-09	1.39283980102267e-06	5.856099
Pola1	-1.88843	1.255316	-9.33798	6.6163803932659e-09	1.50592459794985e-06	5.822197
Rhot2	2.590313	4.177858	9.307182	6.97548916309022e-09	1.57322646042823e-06	5.803209
Colgalt2	-2.62151	-1.30803	-9.28005	7.30861031491627e-09	1.61892244020319e-06	5.790774
Ell3	-3.31791	0.178753	-9.2819	7.28556766843299e-09	1.61892244020319e-06	5.790774
Pth1r	-2.26381	1.906993	-9.22477	8.03968270510022e-09	1.74961831781431e-06	5.757057
Fam111a	-2.4226	0.9435	-9.21663	8.15349159729626e-09	1.75895628728107e-06	5.754745
Prps2	-2.21578	3.333284	-9.1902	8.53507963435282e-09	1.82540336766086e-06	5.738641
Hvcn1	-2.55803	0.425305	-9.14362	9.25339719383207e-09	1.94286533515738e-06	5.711557
Klhl13	-2.21722	0.919104	-9.13954	9.31923797346644e-09	1.94286533515738e-06	5.711557
Rrm2	-3.18143	2.403821	-9.14574	9.21948177948376e-09	1.94286533515738e-06	5.711557
Ly6c1	-2.77344	6.287172	-9.02583	1.13650753975521e-08	2.31111602899892e-06	5.636178
Sult2a7	-4.60391	3.824367	-9.01218	1.16470043980699e-08	2.34919131798143e-06	5.629082
Col1a2	-1.94555	4.04624	-8.99653	1.19642675931928e-08	2.39372189289937e-06	5.620926
St8sia4	-4.12455	-0.55024	-8.96581	1.26309694319309e-08	2.50689376509418e-06	5.600864
Pbk	-4.88914	-0.42066	-8.93374	1.33749292700563e-08	2.63348111318117e-06	5.57947

2) Top 100 differentially-expressed genes in sham + Gln vs. sham – Gln

Symbol	logFC	Avg. expression	t	Crude p	FDR	-logFDR
Kbtbd8	243574	chr6	5.331177	-3.30494	49.13782	4.25770369205409e-22
Lnx1	16924	chr5	6.776497	-2.42657	38.99459	3.59650558575996e-20
Hes5	15208	chr4	8.79475	-2.05083	33.99974	4.97390635733666e-19
Smo	319757	chr6	3.740762	-3.69204	30.19462	4.69522245550957e-18
Tex11	83558	chrX	4.619847	-2.60779	28.25341	1.65462253634392e-17
Defa25	13236	chr8	5.144354	-0.68782	25.96584	8.15502346442779e-17
Jakmip3	74004	chr7	-3.49835	-3.8631	-24.9103	1.78034482504304e-16

Pth2r	213527	chr1	-3.24847	-4.00791	-23.3955	5.77926000290055e-16
Dppa3	73708	chr6	-3.54139	-3.33776	-20.5091	6.73178668447817e-15
Dsg1b	225256	chr18	-2.84033	-4.03523	-18.8138	3.31564637131859e-14
D1Pas1	110957	chr1	2.522246	-3.98581	16.41066	4.02640357623286e-13
Myot	58916	chr18	2.907377	-3.88288	16.39887	4.07918230245929e-13
Mir124a-1hg	268755	chr14	2.465891	-3.96946	15.66344	9.3458311528344e-13
Pdilt	71830	chr7	3.932914	-2.06747	15.66473	9.33259451599667e-13
Txndc12	66073	chr4	4.649093	-2.83529	15.31801	1.39613431427544e-12
Eef1a2	13628	chr2	-2.29447	-1.76489	-15.1934	1.61598705031833e-12
Tigd4	403175	chr3	2.894917	-2.54214	14.62747	3.18610335534463e-12
Ampd3	11717	chr7	2.857464	-1.3076	13.45498	1.39895228212832e-11
Slc6a19os	71390	chr13	2.785087	0.25757	12.74931	3.58912092698182e-11
Hoxa10	15395	chr6	2.351658	-3.77304	12.61681	4.30368490013728e-11
Tnip3	414084	chr6	-4.3359	-2.83694	-11.8065	1.35164295440918e-10
Dock10	210293	chr1	-3.84775	-2.55293	-11.4482	2.28545468923404e-10
Zfyve28	231125	chr5	2.322985	-2.69051	11.36101	2.60189629383393e-10
BC030500	234290	chr8	2.116577	-3.97992	11.34446	2.66700162822638e-10
Myh1	17879	chr11	2.757366	-3.79593	11.20692	3.27848473019942e-10
Rptn	20129	chr3	3.16454	-3.34909	11.10071	3.84999272768062e-10
Sult2a6	629219	chr7	2.948795	-3.73488	10.85915	5.57249862983096e-10
Stac	20840	chr9	4.044652	-2.86852	10.84531	5.69353807034678e-10
Zdhhc15	108672	chrX	3.384296	-3.35337	10.77326	6.36481180797565e-10
Tex19.1	73679	chr11	2.43246	-1.61321	10.65348	7.67100189120029e-10
Zfa-ps	22639	chr10	2.526207	-3.31484	10.167	1.66350193732066e-09
Arl16	70317	chr11	2.016732	-1.79504	10.10763	1.83158475284742e-09
Ccl20	20297	chr1	-1.86698	-1.44897	-9.8243	2.91535082067811e-09
Rsph9	75564	chr17	2.214281	-1.76728	9.425715	5.69523618289506e-09
Adh6a	69117	chr3	-2.20165	-1.46005	-9.08768	1.02000627034304e-08
Utp6	216987	chr11	-1.88763	9.289681	-8.83007	1.60535343828248e-08
Wrn	22427	chr8	2.020132	-3.42425	8.734275	1.90430695730828e-08
Stk4	58231	chr2	-2.79545	-3.32794	-8.26447	4.47528070968981e-08
Fam81b	238726	chr13	3.698145	-2.87844	8.085684	6.24238108298098e-08
Tnfsf13b	24099	chr8	-2.44085	-3.14168	-7.90175	8.82804565067639e-08
Htr1b	15551	chr9	2.815611	-3.12433	7.647796	1.43529879894861e-07
Tnk1	83813	chr11	-2.21772	-2.69393	-7.64285	1.44906338921816e-07
Nme5	75533	chr18	-1.78293	-1.4613	-7.57783	1.6434491367332e-07
D16Ertd519e	52645	chr16	2.678935	-3.47017	7.44236	2.1401458111741e-07
Dlec1	320256	chr9	2.152228	-3.11542	7.265514	3.03233692345982e-07

Prdx6b	320769	chr2	2.373242	-2.98801	6.976687	5.40610998361409e-07
Tdrd6	210510	chr17	1.845123	-0.91997	6.941258	5.80795820162816e-07
Ccdc8	434130	chr7	-2.82064	-3.63806	-6.82335	7.3821175924112e-07
Sv2b	64176	chr7	2.394668	-2.9678	6.770495	8.22500456726879e-07
Zscan12	22758	chr13	2.361872	-2.56117	6.730276	8.93253392383161e-07
Dhx29	218629	chr13	2.125298	-3.1831	6.712437	9.26616385065736e-07
Gria3	53623	chrX	2.71708	-2.97598	6.657721	1.037207581877e-06
Grpr	14829	chrX	1.646155	-2.39725	6.643858	1.06732594798826e-06
Gck	103988	chr11	-2.5064	3.824997	-6.61996	1.12139124457498e-06
Kcnk18	332396	chr19	2.742051	-2.84054	6.618458	1.12488277978774e-06
Pik3c2b	240752	chr1	-2.55441	-2.525	-6.59698	1.17604951805537e-06
Itgb6	16420	chr2	-1.86819	-4.24204	-6.44211	1.62363414454339e-06
Irgm1	15944	chr11	-3.03901	-3.17872	-6.43239	1.65702144360029e-06
Hsd17b6	27400	chr10	1.678645	3.895399	6.416696	1.71238225070305e-06
Ctnna3	216033	chr10	1.905676	-3.55985	6.378442	1.85551066704727e-06
Padi4	18602	chr4	2.159741	-3.28824	6.369094	1.89232648649396e-06
Tcf23	69852	chr5	2.831709	-1.76343	6.317222	2.11073078693084e-06
Angptl1	72713	chr1	2.705238	-2.75401	6.301697	2.18100799663644e-06
Stx19	68159	chr16	1.63465	-4.2371	6.241315	2.47807319944507e-06
Spata16	70862	chr3	1.815354	-2.47371	6.210303	2.64652691845086e-06
Adamts8	30806	chr9	-2.48575	-2.83517	-6.19236	2.74934988407818e-06
Ndufa4l2	407790	chr10	-2.71753	-2.4899	-6.17177	2.8723859588598e-06
Dgke	56077	chr11	2.672937	-2.65391	6.138817	3.08120279557774e-06
Fa2h	338521	chr8	-2.76129	-3.46763	-6.10506	3.31134800167167e-06
Psmd3	22123	chr11	2.664401	-3.48056	6.057769	3.66384307643248e-06
Hoxb7	15415	chr11	-1.5883	0.120801	-6.04014	3.80490084493515e-06
Zkscan8	93681	chr13	2.0918	-2.24938	5.924106	4.88379265557491e-06
Asns	27053	chr6	-2.71099	5.180997	-5.90732	5.06412130383384e-06
Cyp2c39	13098	chr19	-3.49247	-1.9375	-5.86176	5.58954012317481e-06
Tas2r138	387513	chr6	2.11018	-3.16688	5.808422	6.27417898615585e-06
Obp2a	227627	chr2	2.277964	-1.64349	5.729279	7.45400999111526e-06
S100b	20203	chr10	1.928948	-3.23356	5.68561	8.20005910002612e-06
Speer3	71026	chr5	2.342632	-2.4745	5.639252	9.07612784388678e-06
Kcnip4	80334	chr5	2.250014	-2.94238	5.59949	9.90367374740501e-06
Twist1	22160	chr12	2.198852	-3.67251	5.573361	1.04891805018063e-05
Ms4a3	170813	chr19	-2.00444	-1.40354	-5.52774	1.15977550378765e-05
Bsnd	140475	chr4	2.19726	-3.48077	5.449794	1.37769932845919e-05
Tdrd1	83561	chr19	2.047905	-2.64347	5.452017	1.37093728232176e-05

Prg2	19074	chr2	-1.65824	-0.65533	-5.4421	1.40135565642162e-05
Ppp1r1a	58200	chr15	1.685434	-1.20538	5.406414	1.5166859154245e-05
Tmem263	103266	chr10	2.092581	-3.04141	5.399124	1.54141351762713e-05
Ly6a	110454	chr15	-2.40384	3.978126	-5.34616	1.73388649292905e-05
BC037156	494497	chr5	1.835192	-3.55606	5.341614	1.75149357926563e-05
Cldn34b4	619294	chrX	2.359921	-3.30649	5.305498	1.89815561161911e-05
Thrsp	21835	chr7	-2.89662	5.721244	-5.3073	1.89061025508221e-05
Zfpm2	22762	chr15	1.840841	-2.54859	5.308028	1.88748480741045e-05
Actbl2	238880	chr13	2.594106	-2.82534	5.288007	1.97364207344541e-05
Has2	15117	chr15	-2.27987	-3.38145	-5.28275	1.99692200339509e-05
Serpinb3a	20248	chr1	1.988208	-1.57411	5.282986	1.99585412290954e-05
Tas2r114	387346	chr6	1.519788	-4.18812	5.195147	2.42878853474922e-05
Prokr2	246313	chr2	2.105859	-3.30377	5.104493	2.97659896858689e-05
Mmp20	30800	chr9	1.996031	-2.35042	4.965929	4.06778214790692e-05
AU022793	105976	chr15	1.802227	-3.16955	4.937444	4.33840257958994e-05
Trim67	330863	chr8	1.548373	-0.84684	4.917744	4.53619928148738e-05
Piwil2	57746	chr14	2.343965	-2.97679	4.911183	4.60410010878547e-05

3) Top 100 differentially-expressed genes in PHx – Gln vs. sham – Gln

Symbol	logFC	Avg. expression	t	Crude p	FDR	-logFDR
Jakmip3	74004	chr7	-3.40328	-3.8631	-23.9624	3.68953800118122e-16
Pth2r	213527	chr1	-3.21825	-4.00791	-23.0812	7.44535806266426e-16
Dppa3	73708	chr6	-3.91437	-3.33776	-21.8624	2.05124560975452e-15
Dsg1b	225256	chr18	-2.62314	-4.03523	-16.5933	3.29394488272148e-13
Eef1a2	13628	chr2	-2.19792	-1.76489	-13.9354	7.53585407875292e-12
Tnip3	414084	chr6	-5.49472	-2.83694	-13.6351	1.10844609841654e-11
Tnk1	83813	chr11	-3.57902	-2.69393	-13.2257	1.8915152728719e-11
Tnfsf13b	24099	chr8	-3.78443	-3.14168	-12.9045	2.9071878959906e-11
Ccl20	20297	chr1	-2.06048	-1.44897	-11.4539	2.26624782651518e-10
Dock10	210293	chr1	-3.64023	-2.55293	-10.6872	7.27773105085726e-10
Tslp	53603	chr18	-2.96906	-2.74418	-9.96382	2.31628871866898e-09
Stk4	58231	chr2	-3.34039	-3.32794	-9.81926	2.93987269092864e-09
Ly6d	17068	chr15	3.455688	4.68517	9.774461	3.16686677735308e-09
Ndufa4l2	407790	chr10	-3.45632	-2.4899	-7.80601	1.05930823878283e-07
Ranbp17	66011	chr11	-1.8981	-0.61626	-7.66504	1.38833051420793e-07
Ttn	22138	chr2	-2.19265	-1.88335	-7.64981	1.42973234850425e-07
Tbc1d22bos	74092	chr17	-2.20159	-3.75641	-7.29037	2.88665851275031e-07

Amn	93835	chr12	3.202673	-0.03883	6.653061	1.04726781484162e-06
Cxcl9	17329	chr5	-2.19549	2.522625	-6.58407	1.20794685200577e-06
Adamts8	30806	chr9	-2.6072	-2.83517	-6.45217	1.58982891213345e-06
Ms4a3	170813	chr19	-2.21526	-1.40354	-6.35169	1.9628948027535e-06
Zfp85	22746	chr13	-2.74526	-2.14556	-6.2507	2.429321363606e-06
Has2	15117	chr15	-2.58077	-3.38145	-6.16681	2.90284745221192e-06
Gal3st4	330217	chr5	-2.30969	-2.40277	-6.1491	3.01442077442318e-06
Adh6a	69117	chr3	-1.7824	-1.46005	-6.12218	3.19246669513821e-06
Pik3c2b	240752	chr1	-2.43561	-2.525	-6.05664	3.67267191409407e-06
Slc25a31	73333	chr3	-2.41077	-0.13367	-5.94641	4.65439285373339e-06
S100g	12309	chrX	2.263748	-2.41195	5.7691	6.83433880862185e-06
Opn5	353344	chr17	-2.22026	-3.39211	-5.71574	7.67755536541936e-06
Nme5	75533	chr18	-1.58445	-1.4613	-5.6767	8.36141110941082e-06
Ctsg	13035	chr14	-1.93	1.959165	-5.65699	8.73010410919957e-06
Lrtm2	211187	chr6	4.203149	-2.90603	5.419739	1.47421347646323e-05
Fa2h	338521	chr8	-2.51336	-3.46763	-5.25609	2.11933286836123e-05
Nxnl2	75124	chr13	-1.63482	-1.13565	-5.21	2.34934910919056e-05
Esm1	71690	chr13	-2.87149	0.381391	-5.16114	2.6211592328712e-05
Mtnr1a	17773	chr8	2.233235	-1.00103	5.147272	2.70393278302804e-05
Nanos3	244551	chr8	-1.99269	-1.33899	-5.09963	3.00931451292399e-05
Gabrb3	14402	chr7	1.773559	-3.71227	5.046575	3.39098672122358e-05
Prg2	19074	chr2	-1.61764	-0.65533	-5.04452	3.4066868326525e-05
Slc9a2	226999	chr1	2.451346	-2.6716	5.05087	3.35833990648483e-05
Syt2	20980	chr1	-2.02703	-2.63818	-5.01142	3.67072555428388e-05
Grpr	14829	chrX	1.482472	-2.39725	4.846856	5.32692225846368e-05
Mpo	17523	chr11	-2.6694	-1.76992	-4.69353	7.5504821740285e-05
Cyp2c39	13098	chr19	-2.73919	-1.9375	-4.42453	0.00014
Itgb6	16420	chr2	-1.58505	-4.24204	-4.34115	0.000169
Tpsab1	1.01E+08	chr17	-1.98559	-3.46772	-4.34182	0.000169
Tex21	80384	chr12	-1.46792	-0.63179	-4.30096	0.000186
Rec114	73673	chr9	2.612392	0.531803	4.216688	0.000225
Rasl11a	68895	chr5	1.523657	-2.71951	4.161481	0.000256
Cyp2c55	72082	chr19	1.867493	2.824991	4.140035	0.000269
Mybl1	17864	chr1	2.131197	-0.69106	4.114318	0.000285
Rgs16	19734	chr1	-5.3029	3.732226	-3.95378	0.000419
Nwd2	319807	chr5	-1.61648	-0.60395	-3.92459	0.000442
Glul	14645	chr1	1.616919	7.553673	3.896745	0.000471
Grip1	74053	chr10	1.994638	-2.74278	3.813945	0.00057

Sec1	56546	chr7	1.924387	-2.96347	3.730491	0.00069
Lrrc15	74488	chr16	-1.59809	-1.51903	-3.69504	0.000749
Ms4a6b	69774	chr19	-1.69361	-0.32147	-3.52959	0.001094
Bcl6	12053	chr16	-2.32687	-0.22303	-3.50924	0.001147
Clrn2	624224	chr5	-1.63868	-1.9906	-3.30481	0.001827
S100b	20203	chr10	1.55383	-3.23356	3.270452	0.001975
Ppp1r3g	76487	chr13	-2.31689	0.845633	-3.23921	0.00212
Krtap1-3	435273	chr11	-1.93727	-3.44686	-3.17476	0.002452
Slc26a10	216441	chr10	1.521293	-0.56347	3.178087	0.002433
Ctcf	13018	chr8	-1.94913	-3.57803	-3.11137	0.002827
Ugt1a10	394430	chr1	1.663857	2.027515	3.092698	0.002948
Gbp2b	14468	chr3	-1.58118	0.38283	-3.08256	0.003016
AU022793	105976	chr15	1.5086	-3.16955	3.034303	0.00336
Ston2	108800	chr12	1.493977	-2.27106	3.028748	0.003402
Nmnat2	226518	chr1	-1.66189	-4.18977	-2.99672	0.003654
Adgrl1	330814	chr8	-2.01272	5.778141	-2.89616	0.004567
Tgtp2	1E+08	chr11	-1.50805	4.722739	-2.88525	0.004678
Lpl	16956	chr8	1.452535	2.475982	2.836978	0.005203
A1bg	117586	chr15	1.632482	-3.48667	1.061102	0.150993
A1cf	69865	chr19	0.212888	2.987689	0	0.999411
A3galt2	215493	chr4	-0.03409	0.851767	0	0.999997
A4galt	239559	chr15	0.037185	-0.0521	0	1
A4gnt	333424	chr9	0.196021	-1.3789	0	0.999846
AA387883	1E+08	chr19	-0.73398	-0.61955	0	0.800753
AA413626	1E+08	chr11	-0.31894	-1.65751	0	1
AA414768	245350	chrX	0.011091	-0.49812	0	1
AA465934	613254	chr11	0.057664	1.137977	0	1
AA467197	433470	chr2	-0.621	-1.46476	0	0.71565
AA536875	380934	chr14	0.299524	10.5699	0	0.999999
AA543401	1E+08	chr9	-0.11596	2.564105	0	1
AA545190	57866	chr6	-0.09264	-2.19896	0	0.998123
AA619741	241051	chr1	0.049231	2.378333	0	1
AA986860	212439	chr1	-0.18883	0.999102	0	0.951922
Aaas	223921	chr15	0.232216	2.541695	0	1
Aacs	78894	chr5	-1.40523	4.361316	-1.49712	0.075243
Aadac	67758	chr3	0.059055	9.513584	0	0.997482
Aadacl2	639634	chr3	0.358416	-1.38116	0	0.999993
Aadacl3	230883	chr4	0.110694	-0.2696	0	0.999961

Aadat	23923	chr8	0.32114	6.231105	0	0.983043
Aagab	66939	chr9	-0.03435	0.837596	0	1
Aak1	269774	chr6	-0.04399	2.722311	0	0.999989
Aamdc	66273	chr7	-0.25337	3.10299	0	0.999827
Aamp	227290	chr1	0.036914	4.769556	0	0.999986
Aanat	11298	chr11	0.286051	-3.84574	0	0.991742
Aar2	68295	chr2	0.021314	3.54712	0	0.999999

Supplemental Data 7: gene set enrichment analysis (GSEA)

1) M5 curated gene sets by *MSigDB*

https://www.gsea-msigdb.org/gsea/msigdb/mouse/genesets.jsp?collection=M5

2) Top 50 enriched gene sets by GO terms in PHx + Gln vs. PHx – Gln

GO terms	NGenes	Direction	PValue	FDR
GO_MHC_PROTEIN_BINDING	62	Down	1.79226135135738e-06	0.000325032
GO_CELL_DIVISION	563	Down	4.48806214033258e-06	0.000553468
GO_MHC_PROTEIN_COMPLEX	42	Down	5.23635115640165e-06	0.000614031
GO_MHC_CLASS_I_PROTEIN_COMPLEX	32	Down	8.50030691056873e-06	0.00084855
GO_CELLULAR_PROTEIN_COMPLEX_DISASSEMBLY	134	Down	1.07732681798827e-05	0.000933229
GO_CELLULAR_MODIFIED_AMINO_ACID_METABOLIC_PROCESS	217	Down	1.19687852920354e-05	0.000971046
GO_CELLULAR_AMINO_ACID_METABOLIC_PROCESS	352	Down	2.2852182213989e-05	0.00139867
GO_MITOCHONDRIAL_PROTEIN_COMPLEX	129	Down	2.30512971268516e-05	0.00139867
GO_MHC_CLASS_I_PROTEIN_BINDING	54	Down	2.33640997050872e-05	0.00139867
GO_ALPHA_AMINO_ACID_METABOLIC_PROCESS	238	Down	5.35702360673701e-05	0.002359386
GO_MITOTIC_CELL_CYCLE	952	Down	6.5972294809172e-05	0.002766401
GO_TRNA_AMINOACYLATION	50	Down	7.95122171571541e-05	0.00305514
GO_AMINO_ACID_ACTIVATION	50	Down	7.95122171571541e-05	0.00305514
GO_CELLULAR_AMINO_ACID_CATABOLIC_PROCESS	117	Down	0.000161254	0.004458709
GO_SERINE_FAMILY_AMINO_ACID_METABOLIC_PROCESS	43	Down	0.000234915	0.005888164
GO_MITOTIC_CELL_CYCLE_TRANSITION	1309	Down	0.000248248	0.006050184
GO_REGULATION_OF_UBIQUITIN_PROTEIN_LIGASE_ACTIVITY	21	Down	0.000294628	0.006746492
GO_NEGATIVE_REGULATION_OF_CELL_CYCLE_PROCESS	282	Down	0.00030859	0.007021276
GO_MITOTIC_CELL_CYCLE_CHECKPOINT	178	Down	0.000364159	0.007851063
GO_REGULATION_OF_PROTEASOMAL_UBIQUITIN_DEPENDENT_PROTEIN_CATABOLIC_PROCESS	186	Down	0.000374609	0.007977448
GO_CYCLIN_DEPENDENT_PROTEIN_SERINE_THREONINE_KINASE_INHIBITOR_ACTIVITY	14	Down	0.000379641	0.008025514
GO_CELL_CYCLE	1544	Down	0.000596167	0.010864187
GO_PROTEIN_ACTIVATION_CASCADE	87	Down	0.00065283	0.011403249
GO_NEGATIVE_REGULATION_OF_CELL_CYCLE_G1_S_PHASE_TRANSITION	121	Down	0.000697756	0.0119179
GO_CELL_CYCLE_CHECKPOINT	239	Down	0.000706461	0.012000099
GO_PROTEIN_DNA_COMPLEX	93	Down	0.000789036	0.012973855
GO_CELL_CYCLE_G1_S_PHASE_TRANSITION	129	Down	0.00137852	0.018640253
GO_G1_S_TRANSITION_OF_MITOTIC_CELL_CYCLE	129	Down	0.00137852	0.018640253
GO_REGULATION_OF_PROTEIN_CATABOLIC_PROCESS	494	Down	0.001397208	0.018851613
GO_PROTEIN_POLYUBIQUITINATION	275	Down	0.001634063	0.020742808
GO_REGULATION_OF_CELL_CYCLE_CHECKPOINT	38	Down	0.001634863	0.020742808

GO_PROTEIN_TRIMERIZATION	61	Down	0.001785047	0.021796998
GO_PROTEIN_IMPORT	191	Down	0.00178519	0.021796998
GO_PROTEIN_TRANSPORTER_ACTIVITY	104	Down	0.001833792	0.021917476
GO_NEGATIVE_REGULATION_OF_PROTEIN_LOCALIZATION_TO_CELL_PERIPHERY	30	Down	0.001841081	0.021917476
GO_NEGATIVE_REGULATION_OF_PROTEIN_LOCALIZATION_TO_PLASMA_MEMBRANE	30	Down	0.001841081	0.021917476
GO_REGULATION_OF_CELL_CYCLE_PHASE_TRANSITION	403	Down	0.001848376	0.021917476
GO_PROTEIN_LOCALIZATION_TO_CHROMOSOME_CENTROMERIC_REGION	13	Down	0.001882258	0.022231259
GO_REGULATION_OF_CYCLIN_DEPENDENT_PROTEIN_KINASE_ACTIVITY	116	Down	0.001902864	0.022348683
GO_TRANSFERASE_ACTIVITY_TRANSFERRING_ACYL_GROUPS_OTHER_THAN_AMINO_ACYL_GROUPS	203	Down	0.001936669	0.022486272
GO_NEGATIVE_REGULATION_OF_CELLULAR_PROTEIN_LOCALIZATION	195	Down	0.002029439	0.023227519
GO_FATTY_ACID_METABOLIC_PROCESS	1271	Down	0.002102935	0.023835841
GO_SMALL_NUCLEAR_RIBONUCLEOPROTEIN_COMPLEX	62	Down	0.002248523	0.025013232
GO_G_PROTEIN_COUPLED_RECEPTOR_ACTIVITY	487	Up	0.00240494	0.025744552
GO_CELLULAR_LIPID_METABOLIC_PROCESS	1013	Down	0.002488541	0.026184882
GO_NEGATIVE_REGULATION_OF_CELLULAR_PROTEIN_CATABOLIC_PROCESS	84	Down	0.002517965	0.026429495
GO_GLUTAMINE_FAMILY_AMINO_ACID_METABOLIC_PROCESS	13	Down	0.002554586	0.026562523
GO_PROTEIN_CARBOXYLATION	13	Down	0.002554586	0.026562523
GO_IDENTICAL_PROTEIN_BINDING	1524	Down	0.002584368	0.026781868
GO_POSITIVE_REGULATION_OF_CELLULAR_PROTEIN_CATABOLIC_PROCESS	223	Down	0.002626558	0.026992262

3) Top 50 enriched gene sets by GO terms in sham + Gln vs. sham – Gln

GO terms	NGenes	Direction	PValue	FDR
GO_RNA_CATABOLIC_PROCESS	248	Down	1.28231435335461e-09	7.90675030278455e-07
GO_NUCLEAR_TRANSCRIBED_MRNA_CATABOLIC_PROCESS_NONSENSE_MEDIATED_DECAY	132	Down	6.67737477952431e-08	1.52491455150174e-05
GO_PROTEASOMAL_PROTEIN_CATABOLIC_PROCESS	311	Down	3.62617645324056e-05	0.001669
GO_ER_ASSOCIATED_UBIQUITIN_DEPENDENT_PROTEIN_CATABOLIC_PROCESS	69	Down	3.66094970483816e-05	0.001672
GO_RESPONSE_TO_TYPE_I_INTERFERON	109	Down	4.2329474023868e-05	0.001851
GO_NUCLEAR_TRANSCRIBED_MRNA_CATABOLIC_PROCESS_EXONUCLEOLYTIC	33	Down	5.08912957377159e-05	0.00212
GO_MACROMOLECULE_CATABOLIC_PROCESS	1017	Down	5.25788687169201e-05	0.002133
GO_REGULATION_OF_PROTEASOMAL_PROTEIN_CATABOLIC_PROCESS	228	Down	7.04038828505941e-05	0.002569
GO_POSITIVE_REGULATION_OF_CELLULAR_PROTEIN_CATABOLIC_PROCESS	223	Down	7.11094914105386e-05	0.002579
GO_MITOTIC_CELL_CYCLE	952	Down	7.2800964412562e-05	0.002622
GO_REGULATION_OF_PROTEASOMAL_UBIQUITIN_DEPENDENT_PROTEIN_CATABOLIC_PROCESS	186	Down	9.2154326909475e-05	0.003122
GO_REGULATION_OF_CELLULAR_AMINO_ACID_METABOLIC_PROCESS	72	Down	9.30264381077707e-05	0.003134
GO_REGULATION_OF_CELLULAR_PROTEIN_CATABOLIC_PROCESS	328	Down	0.000106	0.003479
GO_CELL_DIVISION	563	Down	0.000114	0.003678
GO_NUCLEAR_TRANSCRIBED_MRNA_CATABOLIC_PROCESS_DEADENYLATION_DEPENDENT_DECAY	63	Down	0.00012	0.003765

GO_REGULATION_OF_DENDRITIC_CELL_DIFFERENTIATION	40	Down	0.000201	0.00554
GO_RESPONSE_TO_INTERLEUKIN_4	50	Down	0.000421	0.009447
GO_NEGATIVE_REGULATION_OF_TYPE_I_INTERFERON_PRODUCTION	54	Down	0.000445	0.009795
GO_CELL_CYCLE	1544	Down	0.000454	0.009833
GO_INTERFERON_GAMMA_MEDIATED_SIGNALING_PATHWAY	114	Down	0.000456	0.009833
GO_RESPONSE_TO_INTERFERON_GAMMA	206	Down	0.000515	0.010498
GO_GLUTATHIONE_DERIVATIVE_BIOSYNTHETIC_PROCESS	21	Down	0.000518	0.010498
GO_GLUTATHIONE_DERIVATIVE_METABOLIC_PROCESS	21	Down	0.000518	0.010498
GO_PROTEIN_CATABOLIC_PROCESS	640	Down	0.000519	0.010498
GO_CELLULAR_RESPONSE_TO_INTERFERON_GAMMA	181	Down	0.000522	0.010523
GO_CELLULAR_CATABOLIC_PROCESS	1489	Down	0.000547	0.010634
GO_POSITIVE_REGULATION_OF_PROTEASOMAL_PROTEIN_CATABOLIC_PROCESS	122	Down	0.000572	0.010979
GO_CELL_CYCLE_PHASE_TRANSITION	353	Down	0.000613	0.011639
GO_REGULATION_OF_TYPE_I_INTERFERON_PRODUCTION	167	Down	0.000645	0.012014
GO_CELL_CYCLE_PROCESS	1309	Down	0.000657	0.012166
GO_CATALYTIC_COMPLEX	1089	Down	0.000828	0.014261
GO_POSITIVE_REGULATION_OF_METAPHASE_ANAPHASE_TRANSITION_OF_CELL_CYCLE	13	Down	0.000987	0.016233
GO_TRNA_AMINOACYLATION	50	Down	0.001013	0.016529
GO_AMINO_ACID_ACTIVATION	50	Down	0.001013	0.016529
GO_CELL_CYCLE_CHECKPOINT	239	Down	0.001232	0.018708
GO_CELL_CYCLE_DNA_REPLICATION	12	Down	0.002333	0.029972
GO_REGULATION_OF_MITOTIC_CELL_CYCLE	620	Down	0.002458	0.031316
GO_MISFOLDED_OR_INCOMPLETELY_SYNTHESIZED_PROTEIN_CATABOLIC_PROCESS	18	Down	0.00266	0.033134
GO_GLUTATHIONE_TRANSFERASE_ACTIVITY	30	Down	0.002677	0.033207
GO_NUCLEOBASE_CONTAINING_SMALL_MOLECULE_INTERCONVERSION	24	Down	0.002722	0.033572
GO_REGULATION_OF_CELL_CYCLE_PHASE_TRANSITION	403	Down	0.002934	0.035263
GO_CATABOLIC_PROCESS	2027	Down	0.003284	0.03835
GO_NEGATIVE_REGULATION_OF_CELL_CYCLE_PROCESS	282	Down	0.003471	0.040304
GO_ENDOPLASMIC_RETICULUM_GOLGI_INTERMEDIATE_COMPARTMENT	151	Down	0.003639	0.041556
GO_POSITIVE_REGULATION_OF_TYPE_I_INTERFERON_PRODUCTION	110	Down	0.003963	0.044028
GO_AMINE_BIOSYNTHETIC_PROCESS	22	Down	0.004226	0.045865
GO_NEGATIVE_REGULATION_OF_MITOTIC_CELL_CYCLE	252	Down	0.004363	0.046709
GO_POSITIVE_REGULATION_OF_CELL_CYCLE_PHASE_TRANSITION	88	Down	0.00461	0.048016
GO_REGULATION_OF_TRANSCRIPTION_INVOLVED_IN_G1_S_TRANSITION_OF_MITOTIC_CELL_CYCLE	32	Down	0.00473	0.049097

3) Top 50 enriched gene sets in PHx – Gln vs. sham – Gln

GO terms	NGenes	Direction	PValue	FDR
GO_INTERFERON_GAMMA_MEDIATED_SIGNALING_PATHWAY	114	Down	7.66836014648068e-10	4.72831086631999e-04
GO_RESPONSE_TO_TYPE_I_INTERFERON	109	Down	1.01012645448066e-07	0.000311
GO_CELLULAR_RESPONSE_TO_INTERFERON_GAMMA	181	Down	4.01056204277532e-06	0.006258
GO_MITOTIC_CELL_CYCLE_CHECKPOINT	178	Down	0.000364	0.007851
GO_CELL_CYCLE	1544	Down	0.000596	0.010864
GO_RESPONSE_TO_INTERFERON_GAMMA	206	Down	1.06986040922546e-05	0.010979
GO_REGULATION_OF_MITOTIC_CELL_CYCLE	620	Down	0.001286	0.017941
GO_G1_S_TRANSITION_OF_MITOTIC_CELL_CYCLE	129	Down	0.001379	0.01864
GO_REGULATION_OF_ALPHA_BETA_T_CELL_PROLIFERATION	50	Down	4.8534064847187e-05	0.025328
GO_CELLULAR_DEFENSE_RESPONSE	96	Down	6.39931980629884e-05	0.02677
GO_RESPONSE_TO_INTERFERON_ALPHA	35	Down	8.06831481669279e-05	0.02677
GO_NEGATIVE_REGULATION_OF_INNATE_IMMUNE_RESPONSE	86	Down	8.24891494186456e-05	0.02677
GO_POSITIVE_REGULATION_OF_MITOTIC_CELL_CYCLE	159	Down	0.004463	0.036841
GO_REGULATION_OF_T_CELL_TOLERANCE_INDUCTION	42	Down	0.000208	0.040535
GO_REGULATION_OF_CELL_CYCLE	1196	Down	0.005785	0.04297
GO_REGULATION_OF_REGULATORY_T_CELL_DIFFERENTIATION	39	Down	0.000318	0.056044
GO_POSITIVE_REGULATION_OF_IMMUNOGLOBULIN_SECRETION	38	Down	0.000361	0.060236
GO_REGULATION_OF_IMMUNOGLOBULIN_SECRETION	44	Down	0.000365	0.060236
GO_WOUND_HEALING_SPREADING_OF_EPIDERMAL_CELLS	31	Up	0.000397	0.061103
GO_POSITIVE_REGULATION_OF_CELL_KILLING	70	Down	0.000416	0.061103
GO_CELL_ADHESION_MEDIATED_BY_INTEGRIN	31	Up	0.000442	0.061434
GO_REGULATION_OF_CYTOKINE_SECRETION_INVOLVED_IN_IMMUNE_RESPONSE	22	Down	0.000578	0.06568
GO_REGULATION_OF_DENDRITIC_CELL_DIFFERENTIATION	40	Down	0.000586	0.06568
GO_REGULATION_OF_T_CELL_MIGRATION	28	Down	0.000929	0.079585
GO_ENDOPLASMIC_RETICULUM_GOLGI_INTERMEDIATE_COMPARTMENT_MEMBRANE	98	Up	0.000993	0.083905
GO_REGULATION_OF_IMMUNOGLOBULIN_PRODUCTION	75	Down	0.001054	0.086614
GO_POSITIVE_REGULATION_OF_IMMUNOGLOBULIN_PRODUCTION	57	Down	0.001181	0.095833
GO_POSITIVE_REGULATION_OF_CELL_JUNCTION_ASSEMBLY	47	Up	0.00129	0.099436
GO_INNATE_IMMUNE_RESPONSE	768	Down	0.001385	0.10236
GO_REGULATION_OF_CELL_KILLING	114	Down	0.001414	0.102577
GO_REGULATION_OF_INTERLEUKIN_4_PRODUCTION	61	Down	0.001466	0.102856
GO_POSITIVE_REGULATION_OF_RHO_PROTEIN_SIGNAL_TRANSDUCTION	35	Up	0.001474	0.102856
GO_CELL_AGGREGATION	42	Up	0.001569	0.104667
GO_MAST_CELL_ACTIVATION	39	Down	0.001588	0.104667
GO_POSITIVE_REGULATION_OF_CYTOKINE_PRODUCTION_INVOLVED_IN_IMMUNE_RESPONSE	80	Down	0.002059	0.119792
GO_NEGATIVE_REGULATION_OF_LEUKOCYTE_MEDIATED_IMMUNITY	108	Down	0.002143	0.120614

GO_REGULATION_OF_INTERFERON_GAMMA_PRODUCTION	166	Down	0.002239	0.123254
GO_NEGATIVE_REGULATION_OF_B_CELL_ACTIVATION	39	Down	0.002352	0.127222
GO_ENDOPLASMIC_RETICULUM_GOLGI_INTERMEDIATE_COMPARTMENT	151	Up	0.002575	0.131203
GO_NEGATIVE_REGULATION_OF_LYMPHOCYTE_MEDIATED_IMMUNITY	95	Down	0.002605	0.131386
GO_REGULATION_OF_NATURAL_KILLER_CELL_MEDIATED_IMMUNITY	78	Down	0.00269	0.131386
GO_POSITIVE_REGULATION_OF_T_CELL_PROLIFERATION	127	Down	0.002723	0.131386
GO_POSITIVE_REGULATION_OF_PRODUCTION_OF_MOLECULAR_MEDIATOR_OF_IMMUNE_RESPONSE	113	Down	0.002732	0.131386
GO_CELLULAR_RESPONSE_TO_VIRUS	29	Down	0.003116	0.141286
GO_MYELOID_LEUKOCYTE_MEDIATED_IMMUNITY	81	Down	0.003214	0.141803
GO_EPITHELIAL_CELL_APOPTOTIC_PROCESS	54	Up	0.003362	0.143006
GO_REGULATION_OF_ALPHA_BETA_T_CELL_ACTIVATION	114	Down	0.003403	0.143006
GO_POSITIVE_REGULATION_OF_ADAPTIVE_IMMUNE_RESPONSE	104	Down	0.003408	0.143006
GO_REGULATION_OF_T_CELL_CHEMOTAXIS	13	Down	0.003409	0.143006
GO_MYELOID_CELL_ACTIVATION_INVOLVED_IN_IMMUNE_RESPONSE	82	Down	0.003652	0.149111

Supplemental Data 8: OAT-positive hepatocytes in the PHx + Gln mice by a whole-slide IHC quantification

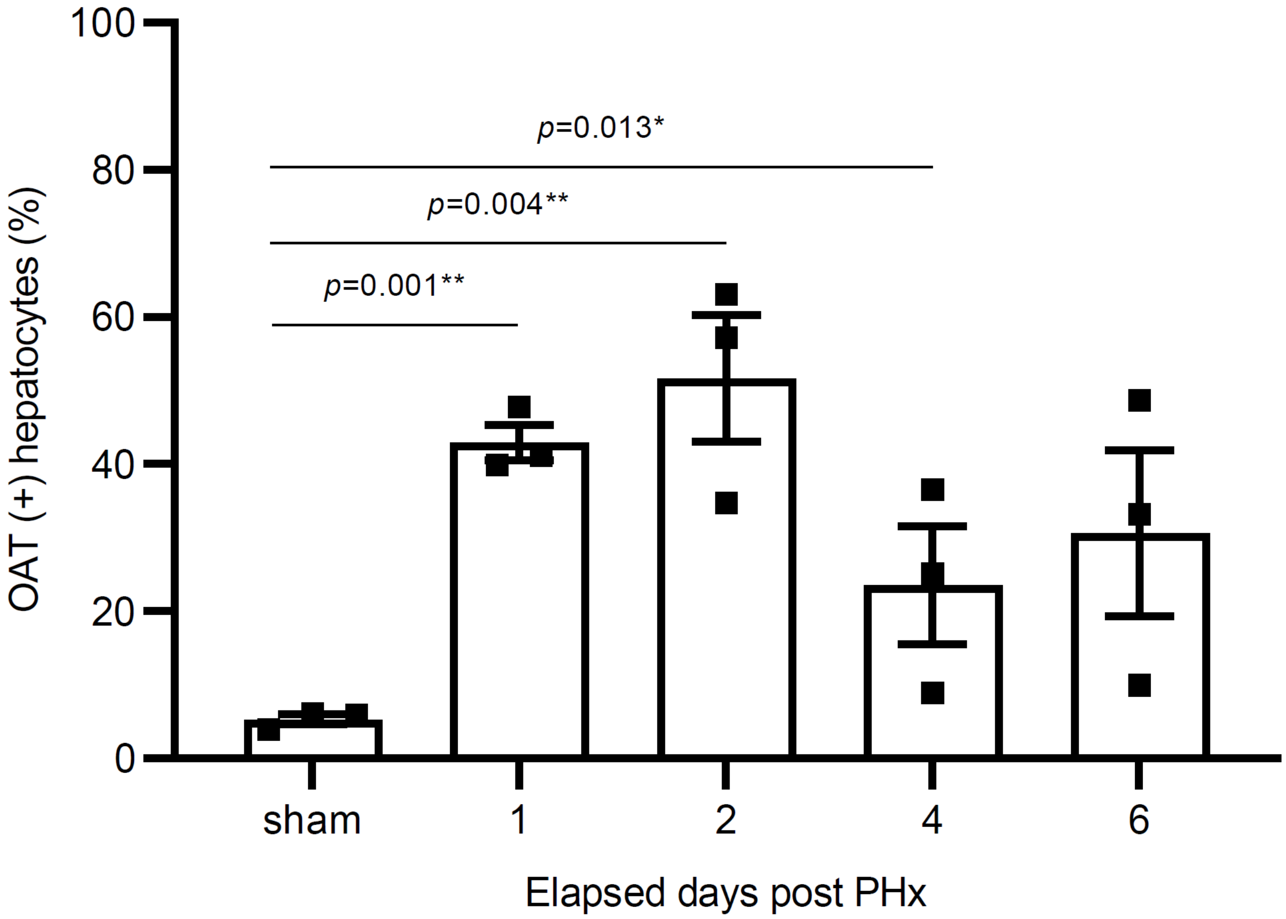

OAT-(+) hepatocytes by a whole-slide IHC quantification (TissueFAXS) in the PHx + Gln mice according to elapsed days post PHx.

Compared by one-way ANOVA. *p<0.05 **p<0.01.

Supplemental Data 9: SHG/TPEF images of liver sections from D0 (sham)-D6

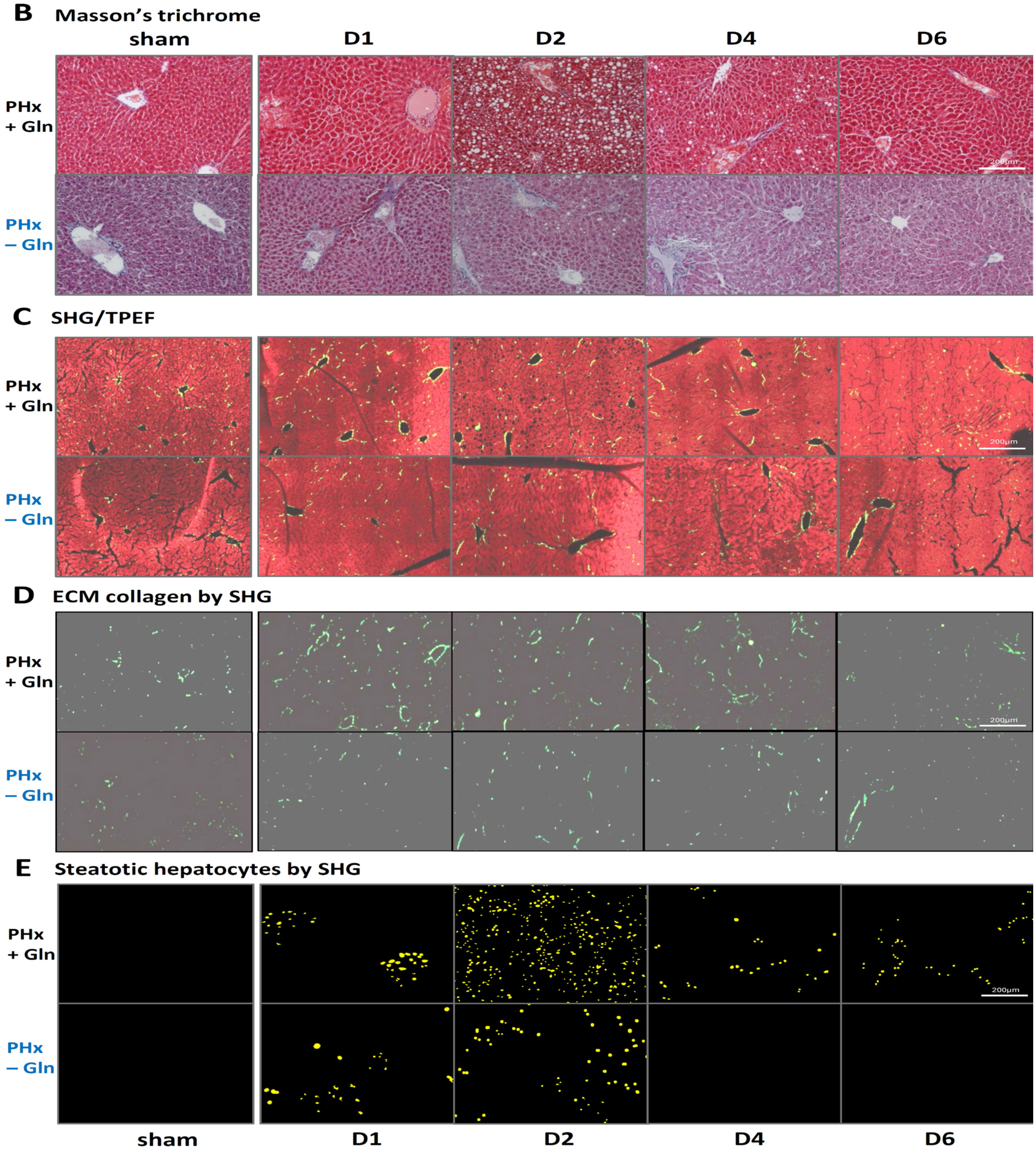

(A) Illustrated diagram of algorithmic approaches in SHG/TPEF. (B) Liver sections stained with Masson's trichrome in PHx + Gln versus PHx – Gln mice. (C) SHG/TPEF merged images of liver sections from PHx + Gln and PHx – Gln mice. (D) SHG showing ECM collagen (green) in liver sections from PHx + Gln vs. PHx – Gln mice. (E) TPEF showing steatotic hepatocytes (yellow) in liver sections from PHx + Gln vs. PHx – Gln mice.
